# PaedVacCOVID - safety of the BNT162b2 vaccine against the SARS-CoV-2 in children with and without comorbidities aged 5 to 11 years

**DOI:** 10.1007/s15010-024-02427-2

**Published:** 2024-11-11

**Authors:** Sarah Holzwarth, Kimiya Saadat, Maximilian Jorczyk, Svenja Dreßen, Sarah Kotsias-Konopelska, Anne Schlegtendal, Christoph Maier, Jochen Schmitt, Kevin Paul, Julia Pagel, Ania C. Muntau, Reinhard Berner, Folke Brinkmann, Nicole Toepfner

**Affiliations:** 1 Department of Pediatrics, Faculty of Medicine, University Hospital Carl Gustav Carus, Technische Universität Dresden, Fetscherstr. 74, 01307 Dresden, Germany; 2 Institute of International Health, Center for Global Health, Charité University Medicine, Augustenburger Platz 1, 13353 Berlin, Germany; 3 University Children’s Hospital, Ruhr-University Bochum, Alexandrinenstraße 5, 44791 Bochum, Germany; 4 Center for Evidence-Based Healthcare (ZEGV), University Hospital Carl Gustav Carus and Carl Gustav Carus Faculty of Medicine, TU Dresden, Fetscherstr. 74, 01307 Dresden, Germany; 5 University Children’s Hospital and German Center of Child and Adolescent Health, University Medical Center Hamburg-Eppendorf, Martinistr. 52, 20251 Hamburg, Germany; 6 German Center for Infection Research (DZIF), Partner Site Hamburg-Lübeck-Borstel-Riems, Inhoffenstr. 7, 38124 Braunschweig, Germany; 7 University Children’s Hospital, Lübeck, Germany; 8 Airway Research Center North (ARCN), Wöhrendamm 80, 22927 Großhansdorf, Germany; 9 German Center for Lung Research (DZL), Großhansdorf, Germany

**Keywords:** SARS-CoV-2, Vaccination, Children, Comorbidity

## Abstract

**Background:**

Little is known about specific safety aspects in children with significant comorbidities receiving the mRNA vaccine BNT162b2, as approval studies did not address this population. This study’s purpose is to evaluate safety and adverse events in these children compared to healthy children.

**Methods:**

In this prospective, multicentre, industry-independent cohort study, caregivers whose children received BNT162b2 were asked to participate in an online questionnaire. Potential side effects were evaluated in ten organ related categories. Frequency of symptoms was compared in both cohorts by bivariate analysis.

**Results:**

From a total of 1,294 responses to the questionnaire, 793 data sets were included into the analysis (179 children with comorbidities and 614 healthy children). Responses were given at a median of 17 days after vaccination. Overall, safety of BNT162b2 was high in both cohorts. Psychological (OR: 3.56, [95% CI: 1.461 to 8.629]), pulmonary (OR: 7.14, [95% CI: 2.039 to 21.48]), gastrointestinal (OR: 2.35, [95% CI: 1.231 to 4.665]), neurological (OR: 1.74, [95% CI: 1.078 to 2.796]) and dermatological (OR: 2.28, [95% CI: 1.220 to 4.172]) side effects were increased in children with comorbidities over healthy controls.

**Conclusion:**

The higher rate of reported post-vaccination symptoms could either be due to a higher susceptibility for symptomatic effects following immune stimulation, or due to a trained awareness to health-related symptoms. The data emphasizes the importance to evaluate safety of the new mRNA COVID-19 vaccines not only in healthy children but also in children with comorbidities. To perform such evaluation should be made mandatory for pharmaceutical enterprises.

**Supplementary Information:**

The online version contains supplementary material available at 10.1007/s15010-024-02427-2.

## Introduction

BNT162b2 was the first vaccine against severe acute respiratory syndrome coronavirus type 2 (SARS-CoV-2) infection that was approved for children [1, 2]. It is a recommended vaccine against coronavirus disease 2019 (COVID-19) for children with comorbidities and immunodeficiency in Germany by the Standing Committee on Vaccination (STIKO) at the Robert Koch-Institute in Berlin, Germany [3] and equally recommended alongside the mRNA-1273 vaccine by the Centers for Disease Control and Prevention (CDC, U. S.) [4]. As of April 2022, 22% of all children in Germany aged 5 to 11 years have received at least one dose of COVID-19 vaccine [5].

Severe adverse events following vaccination with BNT162b2 in children are rare [6, 7] and benefits of COVID-19 vaccination outweigh the risks [8–10]. As adverse events, several cases of myocarditis especially in male adolescents following vaccination [11] as well as single cases of renal failure and pulmonal haemorrhage in children with a rheumatic comorbidity were reported [12]. Overall, the safety profile of BNT162b2 seemed favourable [13, 14].

Throughout the pandemic, it was established that healthy children had few severe courses of SARS-CoV-2 infection and often remained asymptomatic or had a mild, short-term illness [15]. However, compared to their healthy companions, children with comorbidities have a higher rate of severe COVID-19 and COVID-19-associated morbidity and mortality [16, 17]. Therefore, many international guidelines and consensus recommendations recommended vaccination for most children with various comorbidities [18–20]. However, the initial registration study for BNT162b2 included 1517 healthy children aged 5 to 12 years, but children with comorbidities except asthma and obesity were excluded [8]. As vaccine recommendations were specifically addressed for children with comorbidities showing a higher risk for severe COVID-19, this prospective cohort study aimed to systematically evaluate the BNT162b2 safety profiles and side effects in children with significant comorbidities in comparison to healthy children.

## Methods

### Study cohort

The study was designed as an industry-independent, multicentre prospective cohort study to compare the safety and side effects of BNT162b2 vaccine in children aged 5-11 years with and without comorbidities in four German study sides: Department of Pediatrics, University Hospital Carl Gustav Carus, TU Dresden, Hospital of Pediatrics and Adolescent Medicine, Ruhr University, Bochum, Charité University Medicine, Berlin and the Department of Pediatrics, University Medical Center Hamburg-Eppendorf. The study protocol was approved by all respective ethics committees: Dresden and Berlin: BO-EK-578122021 Bochum: 21-7437 and Hamburg: 2022-200298-BO-bet. Written informed consent was obtained from all study participants and/or their legal guardians.

**Fig. 1:**
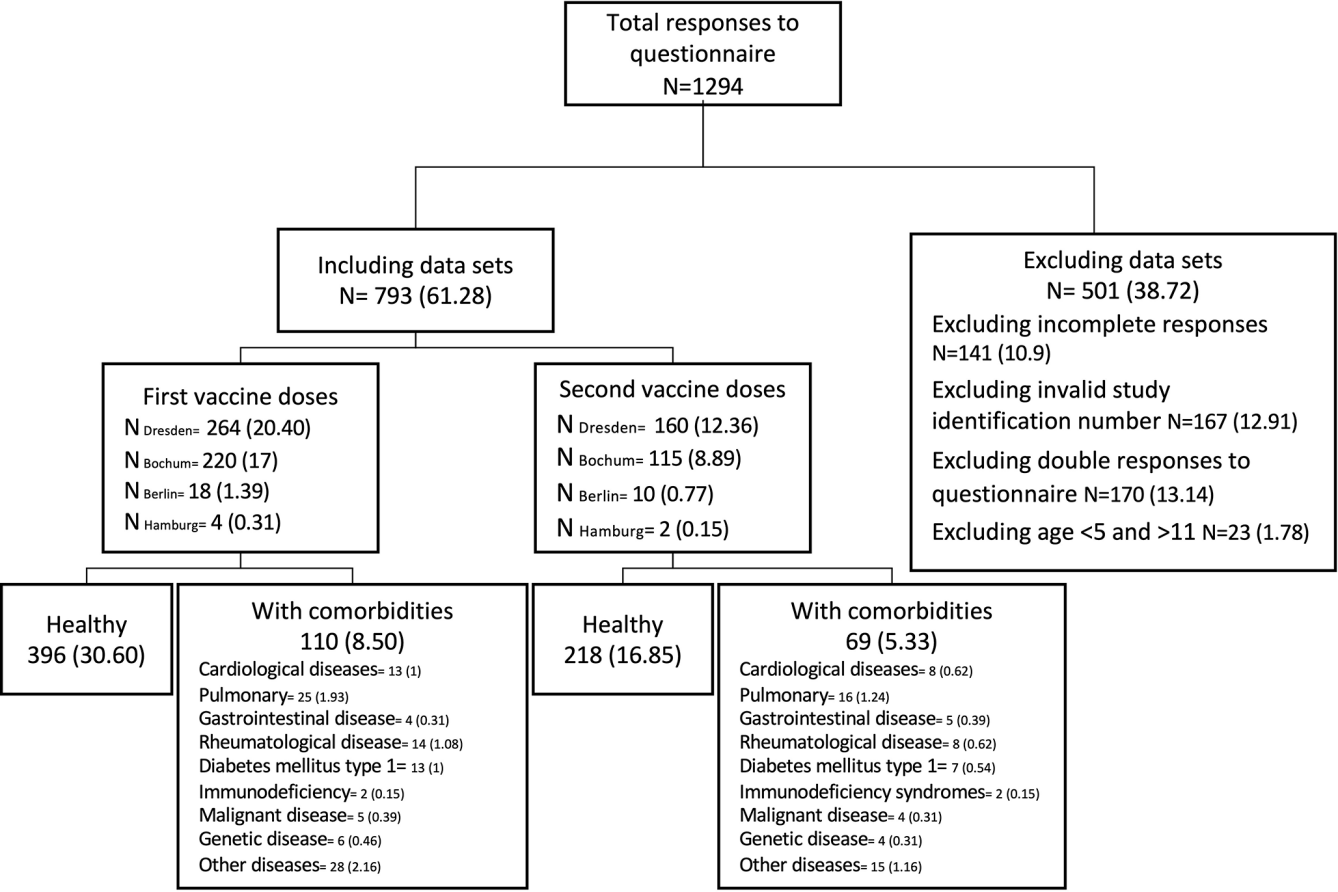
Recruitment (N (%)). All responses to the questionnaire, excluded responses with reasons and responses included for analyses are given. Information on the disease were provided by the parents or caregivers in the online questionnaire. Other diseases included e.g. hyperinsulinemia, allergies, atopic dermatitis, epilepsy, spina bifida, complex congenital anomalies concerning airway and oesophagus, congenital diaphragmatic hernia, OHVIRA, sHLH, diabetes insipidus, Duchenne muscular dystrophy, hypertension (not specified), ADHD, liver transplantation and Dwarfism

Inclusion criteria were age 5–11 years at study enrolement and vaccination with BNT162b2 between December 2021 and March 2022. Exclusion criteria were missing declaration of consent, duplicate or incomplete data in the online survey. Parents and legal guardians were contacted personally and invited to participate in a web-based survey. For the enrolment into the study the parents and legal guardians of the children were provided with the study information and additional age-appropriate versions for children. After informed consent of the children and their parents or legal guardians to study participation, parents and legal guardians were provided with a pseudonymization code, which they could either enter on printed questionnaires as a paper-based survey, or as an access code for the online survey. (Fig. 1) Additional BNT162b2 vaccine doses or SARS-CoV-2 infections could be reported until December 2022, until the online report form was closed and data was extracted in a fully anonymous manner for analysis. The predominant variant of concern at the beginning of the study period was Delta (B.1.617.2), shifting to Omicron (B.1.1.529) at the beginning of 2022 [21]. The antigenic target of the vaccine that the children taking part in this study was based on the original Wuhan SARS-CoV-2 strain.

### Study procedure

The study data was collected using REDCAP electronic data tools. The study was designed to be completed within approximately five minutes in case no side effects were reported. The case report form comprised a general part (demographic data, previous SARS-CoV-2 infections, COVID-19 vaccinations, questions regarding comorbidities and long-term medication). The main section focused on side effects of the previous given COVID-19 vaccination. For children whose caregivers reported a comorbidity a specific part to query COVID-19 vaccination effects on the pre-existing comorbidities was available. Categories queried were local symptoms at the vaccination site, general reactions, skin related symptoms, symptoms regarding the musculoskeletal, pulmonary, nervous, gastrointestinal and cardiovascular system, as well as otolaryngologic or psychological symptoms. Free text information could be provided to obtain precise information regarding the symptoms. In case a single item was selected (e.g. cough) extra questions were asked about the beginning of the symptoms, the end of the symptoms and possible measures that had to be taken due to the symptoms.

### Study outcomes

The primary outcome was the frequency of categorized symptoms reported by the caregivers after vaccination with BNT162b2 compared between healthy children and children with comorbidities. Secondary outcomes where the duration of reported symptoms following vaccination as well as necessary interventions due to these symptoms (e.g. use of analgetics, ambulatory treatment or hospitalization). The time interval between vaccination and onset of symptoms was also surveyed. Hospitalization and mortality were defined as severe adverse events (SAE). Reactions were considered moderate when they required ambulant treatment, mild if no ambulant or stationary treatment was necessary to treat symptoms following vaccination.

### Statistical analysis

Statistical analysis was performed using GraphPad Prism 9. To compare the two variables Fisher exact test was used. The comparison of the side effects in children with and without comorbidities is presented as total numbers, percentages, Odds Ratio (OR) and associated Clopper Pearson two-sided 95% confidence intervals (95%-CI). To control for type I error due to multiple testing, Bonferroni correction controlling for all 64 statistical tests, was performed. A corrected *P* value of ≤ 0.05 was considered significant. All abbreviations are listed in the supplement.

## Results

### Study population

In total, 1294 participant responses were collected. Data of 167 responses were excluded due to invalid study identification numbers, 141 responses were excluded due to incomplete questionnaire data, 170 responses were excluded due to data duplets and data of 23 participants were excluded because their age did not match the inclusion criterion of 5–11 years at the time of the first BNT162b2 vaccine dose. This resulted in 793 data sets which were included in the study analysis comprising 179 responses concerning children with comorbidities and 614 concerning children without comorbidities. The group with comorbidities included 41 vaccinations in children with pulmonary comorbidities, 22 with rheumatologic comorbidities, 21 with cardiological comorbidities, 9 with malignant comorbidities, 4 with primary immunodeficiency, 10 with genetic, 9 with gastrointestinal diseases as well as 20 with type 1 diabetes and 43 with other diseases (Fig. 1 and Table S2, supplement). 506 data sets contained information on the first BNT162b2 vaccine dose, 21.74% of these data sets were from participants with comorbidities. 287 data sets entailed information regarding the second dose of which 24.04% participants had comorbidities. Caregivers of healthy participants completed the online survey at a median of 17 days after the administration of the respective dose, caregivers of children with comorbidities did so after a median of 18 days. The median age in healthy children was 8 years, median height was 135 cm and median weight was 30 kg. The median age in children with comorbidities was 9 years, median height was 132 cm and the median weight was 29 kg (Table S1, supplement). There was no significant difference concerning the age of the two cohort, however children with comorbidities were smaller and lighter.

### Frequency of post-vaccination symptoms

**Fig. 2:**
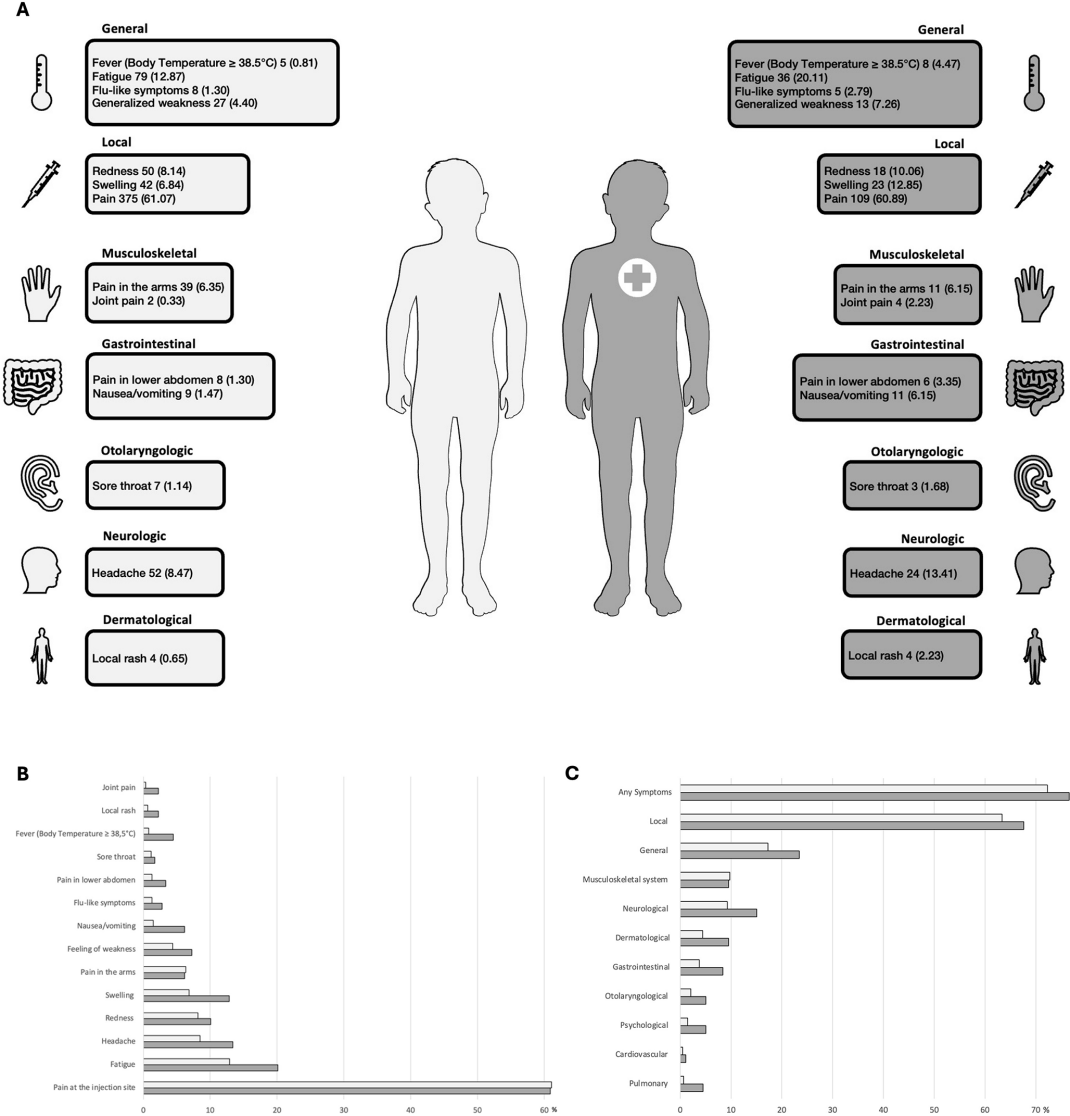
Summarized local and systemic reactions after BNT162b2 vaccination in healthy children and children with comorbidities. (**A**) are the most frequently reported local and systemic post-vaccination symptoms (absolute numbers (relative frequencies in %)) in healthy children (light grey) and children with comorbidities (dark grey with cross) after the first and second dose of BNT162b2 added together. Comorbidities included pulmonary, malignant, rheumatologic, cardiological, genetic and gastrointestinal diseases, as well as primary immunodeficiencies and other diseases. Other reactions included musculoskeletal, gastrointestinal, otolaryngologic, neurologic and dermatologic symptoms. (**B**) depicts the most reported symptoms. (**C**) shows the differences in symptoms by organ category. The Figure was partly generated using Servier Medical Art, provided by Servier, licensed under a Creative Commons Attribution 3.0 unproved license

In total, children with comorbidities and their legal guardians reported certain systemic events and local reactions after vaccination with BNT162b2 more frequently than children who were healthy (Fig. 2). 76.54% of children with comorbidities reported any symptoms at all, whereas for 72.31% of the healthy children any symptoms after vaccination were described. Local reactions were most reported side effects occurring in both cohorts, thereof in 63.36% (389/614) of healthy children and in 67.60% (121/179) of children with comorbidities (OR: 1.21 [95% CI: 0.8534 to 1.724], Table 1). Children with comorbidities reported more swelling on injection site than healthy children (12.85% (23/179) vs. 6.84% (42/614); OR: 2.01 [95% CI: 1.178 to 3.442], Table 2). Pain at the injection site was reported in 61.07% (375/614) of healthy children and 60.89% (109/179) in children with comorbidities and was treated with pain medication in 12 children overall. The local reactions reported lasted on average one to two days. More detailed information is shown in Tables S11 and S21, supplement.

**Table 1:**
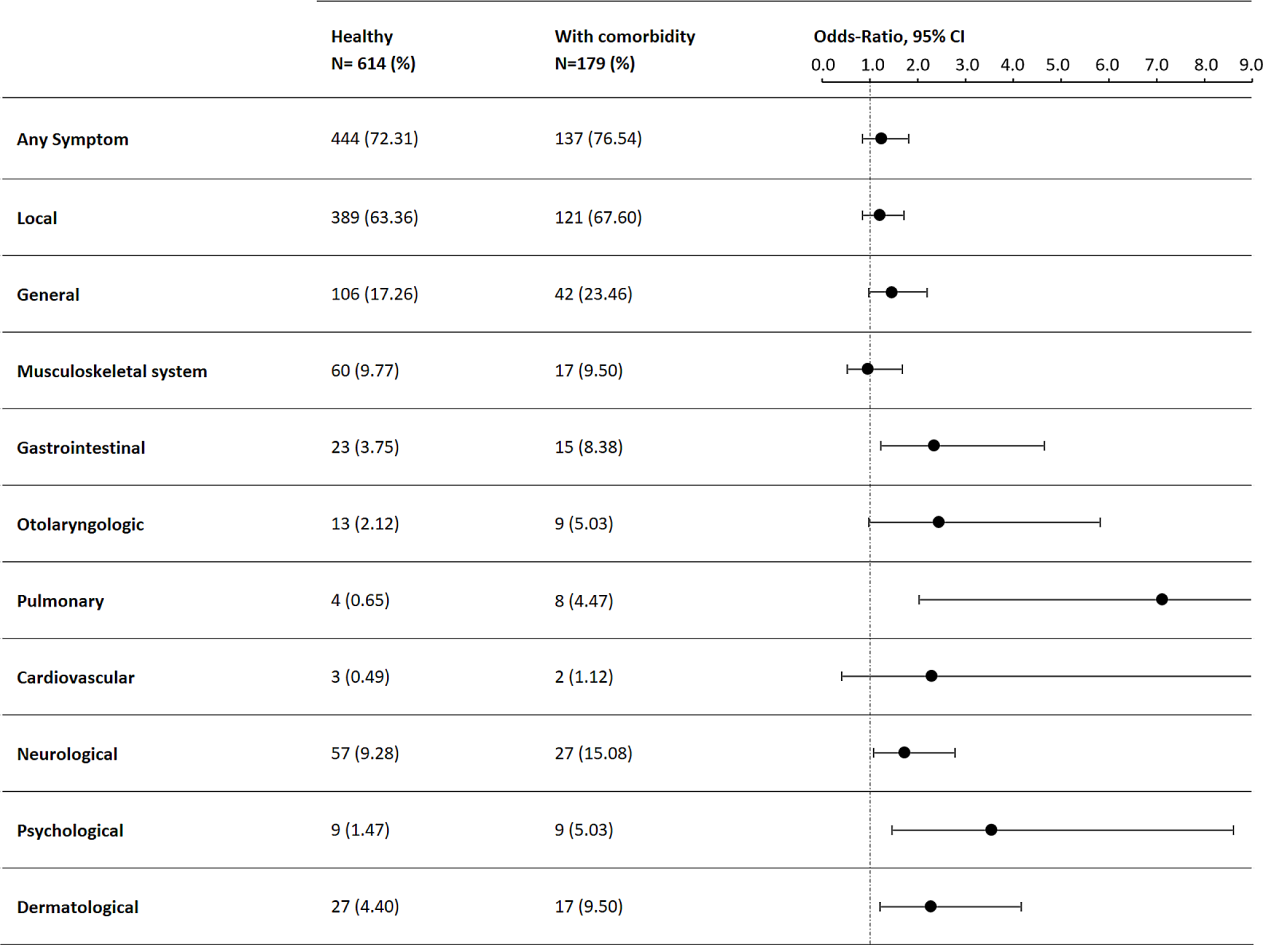
Post-vaccination symptoms in healthy children and children with comorbidities

Children with comorbidities further subdivided into immunocompromising comorbidity and non-immunocompromising comorbidity. Symptoms categorized according to organ systems, reports of first and second dose of BNT162b2 added together. *p ≤ 0.05, **p ≤ 0.01

**Table 2:** Most frequent local and systemic post-vaccination symptoms in healthy children and children with comorbidities

	Healthy *N* = 614 (%)	With comorbidity *N* = 179 (%)	Total *N* = 793 (%)	OR	95% CI	*p*	*p*_correct_
Redness at the injection site	50 (8.14)	18 (10.06)	68 (8.58)	1.261	[0.7095 to 2.226]	0.4482	> 0.9999
Swelling at the injection site	42 (6.84)	23 (12.85)	65 (8.20)	2.008	[1.178 to 3.442]	0.0131 *	0.9694
Pain at the injection site	375 (61.07)	109 (60.89)	484 (61.03)	0.9924	[0.7046 to 1.398]	> 0.9999	> 0.9999
Fever (Body Temperature ≥ 38.5 °C)	5 (0.81)	8 (4.47)	13 (1.64)	5.698	[1.796 to 15.60]	0.0026 **	0.1924
Fatigue	79 (12.87)	36 (20.11)	115 (14.50)	1.705	[1.088 to 2.605]	0.0214 *	> 0.9999
Flu-like symptoms	8 (1.30)	5 (2.79)	13 (1.64)	2.177	[0.7919 to 6.878]	0.1823	> 0.9999
Generalized weakness	27 (4.40)	13 (7.26)	40 (5.04)	1.703	[0.8366 to 3.345]	0.1243	> 0.9999
Pain in the arms	39 (6.35)	11 (6.15)	50 (6.31)	0.9654	[0.4865 to 1.929]	> 0.9999	> 0.9999
Joint pain	2 (0.33)	4 (2.23)	6 (0.76)	6.994	[1.614 to 36.91]	0.0257*	> 0.9999
Pain in lower abdomen	8 (1.30)	6 (3.35)	14 (1.77)	2.627	[0.9002 to 7.726]	0.0989	> 0.9999
Nausea/vomiting	9 (1.47)	11 (6.15)	20 (2.52)	4.401	[1.868 to 10.71]	0.0014 **	0.1036
Sore throat	7 (1.14)	3 (1.68)	10 (1.26)	1.478	[0.4116 to 5.324]	0.7022	> 0.9999
Headache	52 (8.47)	24 (13.41)	76 (9.58)	1.673	[0.9803 to 2.812]	0.0598	> 0.9999
Local rash	4 (0.65)	4 (2.23)	8 (1.01)	3.486	[1.004 to 12.06]	0.0819	> 0.9999

Reports of first and second dose of BNT162b2 added together. *p ≤ 0.05, ** *p* ≤ 0.01

Systemic symptoms were reported in both children with and without comorbidities. Fatigue was the most frequently reported systemic reaction with 12.87% (79/614) in healthy children and 20.11% (36/179) in children with comorbidities (OR: 1.71, [95% CI: 1.088 to 2.605]). Fever above 38.5 °C was reported in 0.81% (5/614) of healthy children and 4.47% (8/179) in children with comorbidities (OR: 5.70, [95% CI: 1.796 to 15.60], Table 2). The average duration of fever was 1.8 days and was treated with antipyretics in 9 of 13 cases. Another leading systemic symptom was generalized weakness in both healthy children and children with comorbidities. This symptom was reported in 4.4% (27/614) of healthy children and 7.26% (13/179) of children with comorbidities. The most common musculoskeletal symptom was pain in the arms, reported in 7.27% (8/110) after the first BNT162b2 dose and 4.35% (3/69) after the second BNT162b2 dose in children with comorbidities and in 6.82% (27/396) of healthy children after first BNT162b2 dose, and in 5.5% (12/218) of healthy children after the second BNT162b2 dose, respectively. Children with comorbidities reported more frequently joint pain (0.33% (2/614) in healthy vs. 2.23% (4/179) in children with comorbidities, OR: 6.99, [95% CI: 1.614 to 36.91], Table 2). In general, musculoskeletal symptoms resolved within 1 to 8 days. Pain medications were used due to pain in the arms and legs, joint pain and swelling and neck and back pain (Tables S13 and S23, supplement).

Children with comorbidities reported more gastrointestinal post-vaccination symptoms (OR: 2.35, [95% CI: 1.231 to 4.665], see Table 1). The leading gastrointestinal symptoms were pain in the upper and lower abdomen, nausea/vomiting and diarrhoea in children with and without comorbidities. Children with comorbidities were 4.4 times more likely to experience nausea and vomiting after receiving a BNT162b2 vaccination than healthy children (OR: 4.40, [95% CI: 1.868 to 10.71]). It was reported in 1.47% of healthy children and 6.15% of children with comorbidities. Pain in the lower abdomen presented in 1.52% (6/396) of healthy children and 2.73% (3/110) of children with comorbidities after the first BNT162b2 dose and 0.92% (2/218), 4.35% (3/69) respectively after second BNT162b2 dose. One healthy child required ambulatory treatment due to pain in the lower abdomen, nausea and vomiting after vaccination. One child with pulmonary comorbidity required ambulatory treatment due to pain in the upper and lower abdomen (Tables S14 and S24, supplement).

The most common otolaryngologic symptom was a sore throat in healthy children as well as in children with comorbidities. The sore throat resolved after an average of 6 days; two healthy children received analgesics as treatment.

Pulmonary symptoms were more frequent after BNT162b2 vaccination in children with comorbidities than in healthy children (OR: 7.14, [95% CI: 2.039 to 21.48]). One healthy child (0.25%) and three children with comorbidities (2.73%) presented with cough after receiving the first BNT162b2 vaccine dose of which one child with comorbidities was treated with both oral medication cough syrup and ß2-mimetic inhalation medication. After the second BNT162b2 vaccine dose 0.92% (2/218) of healthy children and 1.45% (1/69) of children with comorbidities reported cough.

Cardiovascular post-vaccination symptoms were very rarely reported after BNT162b2 vaccinations in both cohorts (0.49% (3/614) in healthy children, 1.12% (2/179) in children with comorbidity). Symptoms reported included chest pain, tachycardia and other non-specified discomforts. Notably, no cases of myocarditis or pericarditis as well as no anaphylaxis were reported.

Headache was the leading neurological symptom and was reported after the first BNT162b2 dose in 8.33% (33/396) of healthy children and in 14.55% (16/110) of children with comorbidities, as well as in 8.72% (19/218) of healthy children and 11.43% (8/69) of children with comorbidities after receiving the second BNT162b2 dose. 2.93% (18/614) of healthy children and 4.47% (8/179) of children with comorbidity received pain medication for headache after BNT162b2 vaccination.

Psychological symptoms after BNT162b2 vaccination were reported more often in children with comorbidities than in healthy children (OR: 3.56, [95% CI: 1.461 to 8.62]). The most frequently reported symptom was sleeping disorder. Parents of children with comorbidities observed sleeping disorders in 2.73% (3/110) of children after the first BNT162b2 dose and in 5.8% (4/69) after the second BNT162b2 dose, whereas only one healthy child was affected after the first BNT162b2 dose (0.25%). Other reported psychological symptoms included concentration problems, hyperactivity and mood swings. One patient reported ongoing sleeping disorders, all other psychological symptoms resolved after a maximum of 17 days. More detailed information is shown in Tables S19 and S29, supplement.

Dermatological symptoms after BNT162b2 vaccination were more frequent in children with comorbidities than in healthy children (OR: 2.28, [95%CI: 1.220 to 4.172]). Lymphadenopathy was reported after the first BNT162b2 dose (1.77%, 7/396) and after the second vaccination (2.29%, 5/218) in healthy children, in no child with comorbidities after the first BNT162b2 dose and in four children (5.8%) after the second BNT162b2 dose. Dermatological symptoms persisted for four days on average, requiring the use of pain medication in two children. Local rashes were seen in no healthy child after the first BNT162b2 vaccine dose and in four healthy children after the second BNT162b2 vaccine dose. Two children with comorbidities showed a local rash after their first dose, whereas three reported on a local rash after dose two of BNT162b2, one required topical dermatological treatment against the local rash, three children with comorbidities required topical treatment against pruritus, dry skin and/or eczema.

No child needed to be hospitalized after BNT162b2 vaccination, no fatalities were reported.

### Frequency of post-vaccination symptoms in children with and without comorbidities after first and second dose

A more detailed analysis regarding post-vaccination symptoms after first and second BNT162b2 doses was performed as displayed in Tables S7 and S8, supplement. Overall, children with comorbidities showed more dermatological symptoms (OR: 4.42, [95% CI: 1.465 to 11.70], p 0.0073) after the second BNT162b2 dose than after the first BNT162b2 dose. Similar results could be seen in healthy children. 2.78% (11/396) reported on dermatological symptoms after the first dose, 7.34% (16/218) after the second dose of BNT162b2. A trend in reported symptoms was also visible for general symptoms (affecting 22 of 110 children with comorbidities after the first BNT162b2 dose and 20 of 69 after the second BNT162b2 dose). Especially, lymphadenopathy was reported in 4 children with comorbidity after the second BNT162b2 dose and in 0 children after the first BNT162b2 dose).

### Differences between immunocompromising and non-immunocompromising comorbidities

When comparing children with comorbidities that compromise the immune system such as rheumatological diseases, malignant diseases and primary immunodeficiencies with children affected by underlying comorbidities that don’t affect the immune system, no significant differences in reported symptoms were seen (*table S31*). However, a trend was detected towards more dermatological symptoms in the immunocompromised group (14.29%, 5/35) than in the group of children with non-immunocompromising comorbidities (8.33%, 12/144).

## Discussion

Vaccination against COVID-19 with mRNA vaccine BNT162b2 was proven safe and effective in preventing severe courses and complications in healthy children aged 5–11 years [2, 8, 22]. However, this vaccination is specifically recommended for children with comorbidities since they are at higher risk for a severe course of COVID-19 requiring hospitalisation [23–25]. As this vulnerable group of children was not included in the initial approval studies [8], this study adds to the safety profiling of BNT162b2 vaccination in children with significant comorbidities based on symptoms reported by the pediatric patients and their legal guardians. In general, this study showed similar results regarding certain side effects of BNT162b2 vaccination when compared to previous reports on SARS-CoV-2 and non-SARS-CoV-2 vaccines [26, 27]. The most common reported post-vaccination symptoms were local side effects such as swelling and redness at the injection site, while there were no reports of severe adverse effects. Children with comorbidities report on more side effects after vaccination with BNT162b2 than healthy children. These side effects include especially gastrointestinal, otolaryngologic, pulmonary, neurological, psychological and dermatological symptoms. For example, children with comorbidities were 4.3 times more likely to experience nausea and vomiting after receiving a BNT162b2 vaccination than healthy children. Also, e.g. fever (defined as measured body temperature ≥ 38.5 °C) was more often reported in children with comorbidities than in healthy children. An underlying pathomechanism behind several side effects has been hypothesized to be a disturbance in the ACE-2/RAAS system by the spike protein produced after vaccination with BNT162b2, including as a result endothelial dysfunction [28]. Also, effects of pro-inflammatory cytokines e.g. leading to altered protein synthesis have been described [29].

The reasons for the striking differences in safety profiles of COVID-19 vaccine between healthy children and children with comorbidities remain unclear. They might even be multiple, as children with a variety of comorbidities and different underlying pathophysiology were included so that further studies are necessary to illuminate the pathological mechanisms leading to the different reported symptoms. One reason might be a potential general increase in susceptibility of the more vulnerable group of patients with comorbidities to symptomatic effects following immune stimulation. Another general mechanism might be a trained awareness of health-related symptoms by legal guardians of children with comorbidities, thus observing their children’s health more closely and detecting as well as reacting to health changes more sensitively, which might lead to overreporting of symptoms. On the other hand, this group may be better trained to assess symptoms and the health status of their children and therefore be more able to differentiate between less worrying symptoms and more serious conditions, resulting in a more solid and reliable reporting of symptoms.

The strength of the study includes a broad study approach taking a random sample of children with comorbidities treated at four different university children’s hospitals in Germany and comparing the findings to simultaneously enrolled age-matched healthy controls. In addition, the study was independent of industrial funding. With a median response time (time difference between completion of online questionnaire and administration of respective dose) under three weeks, there is no significant recall bias, adding another important feature to this analysis. The reports took place during daily routine of the families at flexible timepoints so that the reported symptoms might representatively reflect the experience of legal guardians and their children with BNT162b2 vaccination. Study limitations were the self-reported information of the pediatric patients and their legal guardians without professional medical validation. These self-reports might be biased by one’s individual attention to symptoms. Other study limitations were the varying timepoints of self-reporting comprising the total interval between first and second BNT162b2 dose as well as the missing causal proof of reported symptoms and the effect of BNT162b2 vaccination, as the reports rather reflect a temporal association. It cannot be excluded that due to a limited number of observed vaccinations, less frequently occurring side effects might not have been detected. As part of this study, caregivers were asked to fill out the survey at several time points after the vaccination. If several responses regarding one vaccination were available, the response closest to the vaccination was chosen to enable the symptoms to be reported as accurately as possible. High exclusion rates might have biased the results.

In total, there is only limited data on the safety of mRNA vaccines in adult patients with comorbidities and data on BNT162b2 safety for children with comorbidities are especially lacking. One study reported the safety of BNT162b2 in adult patients with malignancies and found no higher rates of side effects in patients than in healthy controls [30]. However, the study focused on the immunogenicity of the vaccine and evaluated side effects only as secondary outcomes. Other studies focused on the security profile in a small cohort of patients with history of specific allergic reactions without observing an allergic reaction following vaccination with BNT162b2 [31].

Of importance, the increased reported side effects of pediatric patients and their legal guardians were relatively mild and need to be balanced with the increased benefit of BNT162b2 vaccination to avoid severe disease courses of COVID-19 in this vulnerable patient group.

## Conclusion

Overall, the self-reported safety of the vaccine was high in both healthy children and children with comorbidities. However, children with comorbidities showed more adverse events than healthy children. Especially since specific vaccination recommendations for children with comorbidities exist, safety and efficiency of BNT162b2 vaccination in children with comorbidities should further be evaluated. In general, it should be made mandatory for the respective pharmaceutical enterprises to perform studies in children with comorbidities, in particular if it becomes evident that recommendations for vaccination will focus on risk groups.

## Electronic supplementary material

Below is the link to the electronic supplementary material.

Supplementary Material 1

## Data Availability

Data is provided within the manuscript and supplementary material. Further data are available on reasonable request from the authors.
