# Supplementary material for: PaedVacCOVID - safety of the BNT162b2 vaccine against the SARS-CoV-2 in children with and without comorbidities aged 5 to 11 years
Source: Infection. 2024 Nov 11;53(2):615–24. doi: 10.1007/s15010-024-02427-2 (PMC11971181; doi:10.1007/s15010-024-02427-2)
Supplement: Supplementary file 1 — Supplementary Material 1 [file 15010_2024_2427_MOESM1_ESM.docx]

# **Supplement**

**Abbreviations**

BNT162b2 Pfizer-BioNTech COVID-19 Vaccine

CDC Centers for Disease Control and Prevention, Atlanta, United States

CI Confidence interval

COVID-19 Coronavirus Disease 2019

DM1 Type 1 Diabetes mellitus

MIS-C Multisystem Inflammatory Syndrome in Children

mRNA Messenger ribonucleic acid

N Number

OR Odds ratio

PaedVacCOVID Study on Peadiatric Vaccinations against COIVD-19

REDCAP Research Electronic Data Capture (software)

SARS-CoV-2 Severe acute respiratory syndrome coronavirus 2

SD Standard deviation

STIKO Standing Committee on Vaccination at the Robert Koch-Institute in Berlin, Germany

Table S1. Cohort characteristics of participants at baseline

|  | **Healthy** **N=614** | **With comorbidities** **N=179** | **p** | **Total N=793** |
| --- | --- | --- | --- | --- |
| **Age (years) (median (Q1;Q3))** | 8 (7;10) | 9 (7;10) | 0.1875 | 8 (7;10) |
| **Height (cm) (median (Q1;Q3))** | 135 (125;146) | 132 (123;142) | 0.0026 ** | 135 (127;146) |
| **Weight (kg) (median (Q1;Q3))** | 30 (24;36) | 29 (22;34) | 0.0041 ** | 30 (25;36) |
| **Time difference between vaccination and questionnaire response (days) (median (Q1;Q3)** | 17 (10;24) | 18 (12;29) |  | 17 (10;25) |

Table S2. Comorbidities, N (%)

|  | **First vaccine** **N=110 (%)** | **Second vaccine** **N=69 (%)** | **Total** **N= 179 (%)** |
| --- | --- | --- | --- |
| **Cardiological disease** | 13 (11.82) | 8 (11.59) | 21 (11.73) |
| **Pulmonary disease** | 25 (22.73) | 16 (23.19) | 41 (22.91) |
| **Gastrointestinal disease** | 4 (3.64) | 5 (7.25) | 9 (5.03) |
| **Rheumatological disease** | 14 (12.73) | 8 (11.59) | 22 (12.29) |
| **Immunodeficiency** | 2 (1.82) | 2 (2.90) | 4 (2.23) |
| **Malignant disease** | 5 (4.55) | 4 (5.80) | 9 (5.03) |
| **Genetic disorder** | 6 (5.45) | 4 (5.80) | 10 (5.59) |
| **DM 1** | 13 (11.82) | 7 (10.14) | 20 (11.17) |
| **Other disease** | 28 (25.45) | 15 (21.74) | 43 (24.02) |

Table S3. Post-vaccination symptoms in healthy children, children with comorbidities that don’t affect the immune system and immunocompromised comorbidities, further subdivided into rheumatological diseases, primary immunodeficiencies and malignant diseases.

|  |  | **Comorbidities** | | | |  |
| --- | --- | --- | --- | --- | --- | --- |
| **Post-vaccination symptoms** | **Healthy**  **N=614 (%)** | **Immunocompromising comorbidity N=35 (%)** | | | **Non-immuno-compromising comorbidity N=144 (%)** | **Total** **N=793 (%)** |
|  |  | **Rheumatological diseases (22)** | **Primary Immuno-deficiency (4)** | **Malignant diseases (9)** |  |  |
| **Local** | 389 (63.36) | 16 (72.73) | 3 (75.00) | 5 (55.56) | 97 (67.36) | 510 (64.31) |
| **General** | 106 (17.26) | 8 (36.36) | 0 (0.00) | 0 (0.00) | 34 (23.61) | 148 (18.66) |
| **Musculoskeletal system** | 60 (9.77) | 2 (9.09) | 0 (0.00) | 0 (0.00) | 15 (10.42) | 77 (9.71) |
| **Gastrointestinal** | 23 (3.75) | 2 (9.09) | 1 (25.00) | 0 (0.00) | 12 (8.33) | 38 (4.79) |
| **Otolaryngologic** | 13 (2.12) | 0 (0.00) | 1 (25.00) | 0 (0.00) | 8 (5.56) | 22 (2.77) |
| **Pulmonary** | 4 (0.65) | 1 (4.55) | 0 (0.00) | 0 (0.00) | 7 (4.86) | 12 (1.51) |
| **Cardiovascular** | 3 (0.49) | 0 (0) | 0 (0.00) | 0 (0.00) | 2 (1.39) | 5 (0.63) |
| **Neurological** | 57 (9.28) | 5 (22.73) | 1 (25.00) | 0 (0.00) | 21 (14.58) | 84 (10.59) |
| **Psychological** | 9 (1.47) | 1 (4.55) | 0 (0.00) | 0 (0.00) | 8 (5.56) | 18 (2.27) |
| **Dermatological** | 27 (4.40) | 4 (18.18) | 1 (25.00) | 0 (0.00) | 12 (8.33) | 44 (5.55) |

Table S4. Summarized local reactions at the injection site in healthy children and children with comorbidities

|  | **Healthy N=614 (%)** | **With comorbidity N=179 (%)** | **Total** **N=793 (%)** |
| --- | --- | --- | --- |
| **Redness** | 50 (8.14) | 18 (10.06) | 68 (8.58) |
| **Swelling** | 42 (6.84) | 23 (12.85) | 65 (8.20) |
| **Pain** | 375 (61.07) | 109 (60.89) | 484 (61.03) |
| **Other local discomfort** | 8 (1.30) | 4 (2.23) | 12 (1.51) |

Table S5. Summarized systemic post-vaccination symptoms in healthy children and children with comorbidities

|  | **Healthy N=614 (%)** | **With comorbidity N=179 (%)** | **Total** **N=793 (%)** |
| --- | --- | --- | --- |
| **Fever (Body Temperature ≥ 38.5°C)** | 5 (0.81) | 8 (4.47) | 13 (1.64) |
| **Chills** | 9 (1.47) | 3 (1.68) | 12 (1.51) |
| **Hot flashes** | 1 (0.16) | 0 (0.00) | 1 (0.13) |
| **Fatigue** | 79 (12.87) | 36 (20.11) | 115 (14.50) |
| **Flu-like symptoms** | 8 (1.30) | 5 (2.79) | 13 (1.64) |
| **Generalized weakness** | 27 (4.40) | 13 (7.26) | 40 (5.04) |
| **Malaise** | 32 (5.21) | 8 (4.47) | 40 (5.04) |
| **MIS-C** | 0 (0.00) | 0 (0.00) | 0 (0.00) |
| **Other general complaints** | 8 (1.30) | 5 (2.79) | 13 (1.64) |

Table S6**.** Comparator analysis of post-vaccination symptoms in healthy children and children with comorbidities

|  | **Healthy**  **N=614 (%)** | **With comorbidity N=179 (%)** | **OR** | **95% CI** | **p** | **p_corrected_** |
| --- | --- | --- | --- | --- | --- | --- |
| **Local** | 389 (63.36) | 121 (67.60) | 1.207 | [0.8534 to 1.724] | 0.3297 | >0.9999 |
| **General** | 106 (17.26) | 42 (23.46) | 1.469 | [0.9784 to 2.205] | 0.0645 | >0.9999 |
| **Musculoskeletal system** | 60 (9.77) | 17 (9.50) | 0.9689 | [0.5367 to 1.690] | >0.9999 | >0.9999 |
| **Gastrointestinal** | 23 (3.75) | 15 (8.38) | 2.350 | [1.231 to 4.665] | 0.0160 * | >0.9999 |
| **Otolaryngologic** | 13 (2.12) | 9 (5.03) | 2.448 | [0.9779 to 5.835] | 0.0649 | >0.9999 |
| **Pulmonary** | 4 (0.65) | 8 (4.47) | 7.135 | [2.039 to 21.48] | 0.0013 ** | 0.0962 |
| **Cardiovascular** | 3 (0.49) | 2 (1.12) | 2.301 | [0.4052 to 11.33] | 0.3160 | >0.9999 |
| **Neurological** | 57 (9.28) | 27 (15.08) | 1.736 | [1.078 to 2.796] | 0.0374 * | >0.9999 |
| **Psychological** | 9 (1.47) | 9 (5.03) | 3.559 | [1.461 to 8.629] | 0.0091 ** | 0,6734 |
| **Dermatological** | 27 (4.40) | 17 (9.50) | 2.281 | [1.220 to 4.172] | 0.0145 * | >0.9999 |

Table S7**.** Comparator of side effects after first and second vaccination in healthy children

^1^ Not enough data for drawing conclusions

|  | **First Vaccine N= 396 (%)** | **Second Vaccine N= 218 (%)** | **OR** | **95% CI** | **p** | **p_corrected_** |
| --- | --- | --- | --- | --- | --- | --- |
| **Local** | 261 (65.91) | 128 (58.72) | 0.7356 | [0.5207 to 1.028] | 0.0806 | >0.9999 |
| **General** | 74 (18.69) | 32 (14.68) | 0.7486 | [0.4723 to 1.178] | 0.2214 | >0.9999 |
| **Musculoskeletal system** | 45 (11.36) | 15 (6.88) | 0.5764 | [0.3078 to 1.055] | 0.0880 | >0.9999 |
| **Gastrointestinal** | 17 (4.29) | 6 (2.75) | 0.6310 | [0.2560 to 1.549] | 0.3830 | >0.9999 |
| **Otolaryngologic** | 9 (2.27) | 4 (1.83) | 0.8037 | [0.2708 to 2.543] | >0.9999 | >0.9999 |
| **Pulmonary** | 2 (0.51) | 2 (0.92) | 1.824 | [0.2838 to 11.70] | 0.6182 | >0.9999 |
| **Cardiovascular** | 3 (0.76) | 0 (0.00) | NA^1^ | NA^1^ | 0.5559 | >0.9999 |
| **Neurological** | 37 (9.34) | 20 (9.17) | 0.9801 | [0.5611 to 1.722] | >0.9999 | >0.9999 |
| **Psychological** | 7 (1.77) | 2 (0.92) | 0.5146 | [0.1073 to 2.316] | 0.5027 | >0.9999 |
| **Dermatological** | 11 (2.78) | 16 (7.34) | 2.772 | [1.256 to 6.342] | 0.0124 * | 0.9176 |

Table S8**.** Comparator of side effects after first and second vaccination in children with comorbidities

^1^ Not enough data for drawing conclusions

|  | **First Vaccine N=110 (%)** | **Second Vaccine N=69 (%)** | **OR** | **95% CI** | **p** | **p_corrected_** |
| --- | --- | --- | --- | --- | --- | --- |
| **Local** | 74 (67.27) | 47 (68.12) | 1.039 | [0.5508 to 1.925] | >0.9999 | >0.9999 |
| **General** | 22 (20.00) | 20 (28.99) | 1.633 | [0.7958 to 3.308] | 0.2050 | >0.9999 |
| **Musculoskeletal system** | 12 (10.91) | 5 (7.25) | 0.6380 | [0.2400 to 1.910] | 0.6014 | >0.9999 |
| **Gastrointestinal** | 9 (8.18) | 6 (8.70) | 1.069 | [0.3582 to 3.041] | >0.9999 | >0.9999 |
| **Otolaryngologic** | 5 (4.55) | 4 (5.80) | 1.292 | [0.3845 to 4.511] | 0.7352 | >0.9999 |
| **Pulmonary** | 5 (4.55) | 3 (4.35) | 0.9545 | [0.2462 to 3.775] | >0.9999 | >0.9999 |
| **Cardiovascular** | 2 (1.82) | 0 (0.00) | NA^1^ | NA^1^ | 0.5236 | >0.9999 |
| **Neurological** | 19 (17.27) | 8 (11.59) | 0.6281 | [0.2689 to 1.453] | 0.3920 | >0.9999 |
| **Psychological** | 5 (4.55) | 4 (5.80) | 1.292 | [0.3845 to 4.511] | 0.7352 | >0.9999 |
| **Dermatological** | 5 (4.55) | 12 (17.39) | 4.421 | [1.465 to 11.70] | 0.0073** | 0.5402 |

Table S9**.** Comparator of side effects after first vaccination in children with and without comorbidities

|  | **First Vaccine -healthy N=396 (%)** | **First Vaccine - with comorbidity N=110 (%)** | **OR** | **95% CI** | **p- value** | **p_corrected_** |
| --- | --- | --- | --- | --- | --- | --- |
| **Local** | 261 (65.91) | 74 (67.27) | 1.063 | [0.6751 to 1.658] | 0.8206 | >0.9999 |
| **General** | 74 (18.69) | 22 (20.00) | 1.088 | [0.6395 to 1.869] | 0.7837 | >0.9999 |
| **Musculoskeletal system** | 45 (11.36) | 12 (10.91) | 0.9551 | [0.4979 to 1.868] | >0.9999 | >0.9999 |
| **Gastrointestinal** | 17 (4.29) | 9 (8.18) | 1.987 | [0.8250 to 4.658] | 0.1389 | >0.9999 |
| **Otolaryngologic** | 9 (2.27) | 5 (4.55) | 2.048 | [0.7533 to 5.963] | 0.1978 | >0.9999 |
| **Pulmonary** | 2 (0.51) | 5 (4.55) | 9.381 | [1.974 to 47.43] | 0.0065 ** | 0,481 |
| **Cardiovascular** | 3 (0.76) | 2 (1.82) | 2.426 | [0.4252 to 11.98] | 0.2984 | >0.9999 |
| **Neurological** | 37 (9.34) | 19 (17.27) | 2.026 | [1.091 to 3.603] | 0.0250 * | >0.9999 |
| **Psychological** | 7 (1.77) | 5 (4.55) | 2.646 | [0.9343 to 7.699] | 0.1463 | >0.9999 |
| **Dermatological** | 11 (2.78) | 5 (4.55) | 1.667 | [0.6303 to 4.962] | 0.3581 | >0.9999 |

Table S10**.** Comparator of side effects after second vaccination in children with and without comorbidities

^1^ Not enough data for drawing conclusions

|  | **Second Vaccine - healthy N=218 (%)** | **Second Vaccine -**  **with comorbidity N=69 (%)** | **OR** | **95% CI** | **p- value** | **p_corrected_** |
| --- | --- | --- | --- | --- | --- | --- |
| **Local** | 128 (58.72) | 47 (68.12) | 1.502 | [0.8492 to 2.607] | 0.2025 | >0.9999 |
| **General** | 32 (14.68) | 20 (28.99) | 2.372 | [1.276 to 4.439] | 0.0113 * | 0.8362 |
| **Musculoskeletal system** | 15 (6.88) | 5 (7.25) | 1.057 | [0.4090 to 2.807] | >0.9999 | >0.9999 |
| **Gastrointestinal** | 6 (2.75) | 6 (8.70) | 3.365 | [1.010 to 11.12] | 0.0422* | >0.9999 |
| **Otolaryngologic** | 4 (1.83) | 4 (5.80) | 3.292 | [0.9321 to 11.51] | 0.0980 | >0.9999 |
| **Pulmonary** | 2 (0.92) | 3 (4.35) | 4.909 | [0.9785 to 27.93] | 0.0918 | >0.9999 |
| **Cardiovascular** | 0 (0.00) | 0 (0.00) | NA^1^ | NA^1^ | >0.9999 | >0.9999 |
| **Neurological** | 20 (9.17) | 8 (11.59) | 1.298 | [0.5709 to 3.122] | 0.6413 | >0.9999 |
| **Psychological** | 2 (0.92) | 4 (5.80) | 6.646 | [1.508 to 35.27] | 0.0313 * | >0.9999 |
| **Dermatological** | 16 (7.34) | 12 (17.39) | 2.658 | [1.158 to 5.750] | 0.0198 * | >0.9999 |

## **Post-vaccination symptoms healthy**

Table S11. Local post-vaccination symptoms in healthy children

|  | | **Redness at the injection site** | **Swelling at the injection site** | **Pain at the injection site** | **Other local discomfort** |
| --- | --- | --- | --- | --- | --- |
| **After First vaccination** | | 33/396 (8.33) | 28/396 (7.07) | 250/396 (63.13) | 4/396 (1.01) |
| **After Second vaccination** | | 17/218 (7.80) | 14/218 (6.42) | 125/218 (57.34) | 4/218 (1.83) |
| **Beginning & Duration** | |  |  |  |  |
| **Beginning (days after vaccination, mean ± SD/N)** | 0.41±0.54/ 49 | 0.34±0.47/ 40 | 0.29±0.48/ 373 | 1.13±2.03/ 8 |  |
| **Duration (days, mean ± SD/N)** | 2.14±1.41/ 49 | 2.38±1.15/ 40 | 1.92±1.02/ 373 | 1.75±1.00/ 8 |  |
| **Aftermath** | |  |  |  |  |
| **Pain Medication** |  |  | 9/614 (1.47) |  |  |
| **Ambulatory** | 0/614 (0) | 0/614 (0) | 0/614 (0) | 0/614 (0) |  |
| **Inpatient** | 0/614 (0) | 0/614 (0) | 0/614 (0) | 0/614 (0) |  |
| **Mortality** | 0/614 (0) | 0/614 (0) | 0/614 (0) | 0/614 (0) |  |
| **Other** | 1/614 (0.16) | 3/614 (0.49) | 4/614 (0.65) | 1/614 (0.16) |  |

Table S12. General post-vaccination symptoms in healthy children. PIMS = Pediatric inflammatory multisystem syndrome

|  | | **Fever**  **(Body Temperature**  **≥ 38.5°C)** | **Chills** | **Hot flashes** | **Fatigue** | **Flu- like symptoms** | **Generalized weakness** | **Malaise** | **MIS-C** | **Other general complaints** |
| --- | --- | --- | --- | --- | --- | --- | --- | --- | --- | --- |
| **After First vaccination** | | 2/396 (0.51) | 4/396 (1.01) | 1/396 (0.25) | 57/396 (14.39) | 5/396 (1.26) | 20/396 (5.05) | 17/396 (4.29) | 0/396 (0) | 7/396 (1.77) |
| **After Second vaccination** | | 3/218 (1.38) | 5/218 (2.29) | 0/218 (0) | 22/218 (10.09) | 3/218 (1.38) | 7/218 (3.21) | 15/218 (6.88) | 0/218 (0) | 1/218 (0.46) |
| **Beginning & Duration** | |  |  |  |  |  |  |  |  |  |
| **Beginning (days after vaccination, mean ± SD/N)** | 2.20±1.10/5 | 0.56±0.73/ 9 | 0±0/ 1 | 0.54±0.75/ 79 | 2.25±1.16/ 8 | 0.56±0.75/ 27 | 0.72±0.85/ 32 | 0±0/ 1 | 2.25±3.37/ 8 |  |
| **Duration (days, mean ± SD/N)** | 1.5±1.41/ 5 | 0.89±0.55/ 9 | 2±0/ 1 | 1.16±0.64/ 79 | 3.25±2.55/ 8 | 1.35±0.83/ 27 | 1.69±1.44/ 32 | 0±0/ 1 | 1.69±0.96/ 8 |  |
| **Aftermath** | |  |  |  |  |  |  |  |  |  |
| **Analgesics/Pain medication** | 4/614 (0.65) | 3/614 (0.49) | 0/614 (0) |  | 5/614 (0.81) |  | 7/614 (1.14) | 0/614 (0) | 0/614 (0) |  |
| **Ambulatory** | 0/614 (0) | 0/614 (0) | 0/614 (0) | 0/614 (0) | 0/614 (0) | 1/614 (0.16) | 0/614 (0) | 0/614 (0) | 1/614 (0.16) |  |
| **Inpatient** | 0/614 (0) | 0/614 (0) | 0/614 (0) | 0/614 (0) | 0/614 (0) | 0/614 (0) | 0/614 (0) | 0/614 (0) | 0/614 (0) |  |
| **Mortality** | 0/614 (0) | 0/614 (0) | 0/614 (0) | 0/614 (0) | 0/614 (0) | 0/614 (0) | 0/614 (0) | 0/614 (0) | 0/614 (0) |  |
| **Other** | 0/614 (0) | 0/614 (0) | 0/614 (0) | 0/614 (0) | 0/614 (0) | 0/614 (0) | 0/614 (0) | 0/614 (0) | 0/614 (0) |  |

Table S13. Musculoskeletal post-vaccination symptoms in healthy children

|  | | **Muscle weakness** | **Muscle Pain** | **Muscle twitching** | **Neck/back pain** | **Pain in the arms** | **Pain in the legs** | **Joint pain** | **Joint swelling** | **Pain in limbs** | **Other complaints** |
| --- | --- | --- | --- | --- | --- | --- | --- | --- | --- | --- | --- |
| **After First vaccination** | | 2/396 (0.51) | 15/396 (3.79) | 0/396 (0) | 2/396 (0.51) | 27/396 (6.82) | 6/396 (1.52) | 2/396 (0.51) | 0/396 (0) | 7/396 (1.77) | 1/396 (0.25) |
| **After Second vaccination** | | 0/218 (0) | 0/218 (0) | 0/218 (0) | 2/218 (0.92) | 12/218 (5.5) | 0/218 (0) | 0/218 (0) | 0/218 (0) | 0/218 (0) | 1/218 (0.46) |
| **Beginning & Duration** | |  |  |  |  |  |  |  |  |  |  |
| **Beginning (days after vaccination, mean ± SD/N)** | 2.5±0.71/ 2 | 0.53±0.99/ 15 | 0±0/ 0 | 1.75±2.87/ 4 | 0.44±0.64/ 39 | 2.5±1.38/ 6 | 1±1.41/ 2 | 0±0/ 0 | 1.67±1.63/ 6 | 1±0/ 1 |  |
| **Duration (days, mean ± SD/N)** | 3.5±2.12/ 2 | 2.47±1.73/ 15 | 0±0/ 0 | 2.25±1.89/ 4 | 1.92±1.04/ 38 | 2.2±1.64/ 5 | 1±0/2 | 0±0/ 0 | 1.83±1.60/ 6 | 3±0/ 1 |  |
| **Aftermath** | |  |  |  |  |  |  |  |  |  |  |
| **Pain medication** |  | 0/614 (0) | 0/614 (0) | 1/614 (0.16) | 1/614 (0.16) | 3/614 (0.49) | 1/614 (0.16) | 0/614 (0) | 2/614 (0.33) | 0/614 (0) |  |
| **Ambulatory** | 0/614 (0) | 0/614 (0) | 0/614 (0) | 0/614 (0) | 0/614 (0) | 0/614 (0) | 0/614 (0) | 0/614 (0) | 0/614 (0) | 0/614 (0) |  |
| **Inpatient** | 0/614 (0) | 0/614 (0) | 0/614 (0) | 0/614 (0) | 0/614 (0) | 0/614 (0) | 0/614 (0) | 0/614 (0) | 0/614 (0) | 0/614 (0) |  |
| **Mortality** | 0/614 (0) | 0/614 (0) | 0/614 (0) | 0/614 (0) | 0/614 (0) | 0/614 (0) | 0/614 (0) | 0/614 (0) | 0/614 (0) | 0/614 (0) |  |
| **Other** | 0/614 (0) | 1/614 (0.16) | 0/614 (0) | 0/614 (0) | 0/614 (0) | 1/614 (0.16) | 0/614 (0) | 0/614 (0) | 0/614 (0) | 0/614 (0) |  |

Table S14. Gastrointestinal post-vaccination symptoms in healthy children

|  | | **Pain in upper abdomen** | **Pain in lower abdomen** | **Nausea/vomiting** | **Obstipation** | **Diarrhea** | **Other stool changes** | **Unwanted weight changes** | **Other complaints** |
| --- | --- | --- | --- | --- | --- | --- | --- | --- | --- |
| **After First vaccination** | | 4/396 (1.01) | 6/396 (1.52) | 6/396 (1.52) | 1/396 (0.25) | 5/396 (1.26) | 1/396 (0.25) | 0/396 (0) | 1/396 (0.25) |
| **After Second vaccination** | | 1/218 (0.46) | 2/218 (0.92) | 3/218 (1.38) | 0/218 (0) | 0/218 (0) | 1/218 (0.46) | 0/218 (0) | 0/218 (0) |
| **Beginning & Duration** | |  |  |  |  |  |  |  |  |
| **Beginning (days after vaccination, mean ± SD/N)** | 1.4±1.52/ 5 | 3.38±4.9/ 8 | 2.33± 3.12/ 9 | 5± 0/ 1 | 7±6.98/ 4 | 5± 2.83/ 2 | 0±0/ 0 | 7± 0/ 1 |  |
| **Duration (days, mean ± SD/N)** | 0.9±0.65/ 5 | 2.25±2.1/ 8 | 1.68± 1.79/ 8 | 3± 0/ 1 | 3.75±1.89/ 4 | 1.75± 1.77/ 2 | 0±0/ 0 | 14± 0/ 1 |  |
| **Aftermath** | |  |  |  |  |  |  |  |  |
| **Pain Medication** | 0/614 (0) | 1/614 (0.16) | 3/614 (0.49) | 0/614 (0) | 0/614 (0) | 0/614 (0) |  |  |  |
| **Antiemetics** | 1/614 (0.16) | 0/614 (0) | 1/614 (0.16) | 0/614 (0) | 2/614 (0.33) | 0/614 (0) | 0/614 (0) | 0/614 (0) |  |
| **Ambulatory** | 0/614 (0) | 1/614 (0.16) | 1/614 (0.16) | 0/614 (0) | 0/614 (0) | 0/614 (0) | 0/614 (0) | 0/614 (0) |  |
| **inpatient** | 0/614 (0) | 0/614 (0) | 0/614 (0) | 0/614 (0) | 0/614 (0) | 0/614 (0) | 0/614 (0) | 0/614 (0) |  |
| **mortality** | 0/614 (0) | 0/614 (0) | 0/614 (0) | 0/614 (0) | 0/614 (0) | 0/614 (0) | 0/614 (0) | 0/614 (0) |  |
| **Other** | 0/614 (0) | 0/614 (0) | 1/614 (0.16) | 0/614 (0) | 0/614 (0) | 0/614 (0) | 0/614 (0) | 0/614 (0) |  |

Table S15. Otolaryngologic post-vaccination symptoms in healthy children

|  | | **Nose-bleed** | **Redness of the oral mucosa** | **Swelling of the tongue** | **Swelling of the lips** | **Discomfort in the mouth** | **Toothache** | **Bleeding of the gums** | **Tightness in the throat** | **Sore throat** | **Earache** | **Hoarse-ness** | **Facial swelling** | **Swollen lymph nodes** | **Painful lymph nodes** | **Olfactory disorder** | **Taste disorder** | **Other complaints** |
| --- | --- | --- | --- | --- | --- | --- | --- | --- | --- | --- | --- | --- | --- | --- | --- | --- | --- | --- |
| **After First vaccination** | | 2/396 (0.51) | 0/396 (0) | 0/396 (0) | 0/396 (0) | 1/396 (0.25) | 0/396 (0) | 0/396 (0) | 0/396 (0) | 4/396 (1.01) | 2/396 (0.51) | 0/396 (0) | 0/396 (0) | 1/396 (0.25) | 0/396 (0) | 0/396 (0) | 0/396 (0) | 2/396 (0.51) |
| **After Second vaccination** | | 0/218 (0) | 0/218 (0) | 0/218 (0) | 0/218 (0) | 0/218 (0) | 0/218 (0) | 0/218 (0) | 0/218 (0) | 3/218 (1.38) | 1/218 (0.46) | 1/218 (0.46) | 0/218 (0) | 0/218 (0) | 0/218 (0) | 0/218 (0) | 0/218 (0) | 1/218 (0.46) |
| **Beginning & Duration** | |  |  |  |  |  |  |  |  |  |  |  |  |  |  |  |  |  |
| **Beginning (days after vaccination, mean ±SD/N)** | 6± 5.66/ 2 | 0±0/ 0 | 0±0/ 0 | 0±0/ 0 | 3± 0/ 1 | 0±0/ 0 | 0±0/ 0 | 0±0/ 0 | 3.85±2.04 /7 | 7.33± 6.81/ 3 | 4± 0/ 1 | 0±0/ 0 | 3± 0/ 1 | 0±0/ 0 | 0±0/ 0 | 0±0/ 0 | 4.33± 2.31/ 3 |  |
| **Duration (days, mean ±SD/N)** | 1.75± 1.77/ 2 | 0±0/ 0 | 0±0/ 0 | 0±0/ 0 | 8± 0/ 1 | 0±0/ 0 | 0±0/ 0 | 0±0/ 0 | 5.21±4.54 /7 | 2.33± 2.31/ 3 | 1± 0/ 1 | 0±0/ 0 | 8± 0/ 1 | 0±0/ 0 | 0±0/ 0 | 0±0/ 0 | 7.33± 5.86/ 3 |  |
| **Aftermath** | |  |  |  |  |  |  |  |  |  |  |  |  |  |  |  |  |  |
| **Pain Medication** |  |  |  |  |  | 0/614 (0) | 0/614 (0) | 0/614 (0) | 2/614 (0.33) | 3/614 (0.49) | 0/614 (0) |  | 0/614 (0) | 0/614 (0) |  |  | 0/614 (0) |  |
| **Ambulatory** | 0/614 (0) | 0/614 (0) | 0/614 (0) | 0/614 (0) | 0/614 (0) | 0/614 (0) | 0/614 (0) | 0/614 (0) | 0/614 (0) | 0/614 (0) | 0/614 (0) | 0/614 (0) | 0/614 (0) | 0/614 (0) | 0/614 (0) | 0/614 (0) | 0/614 (0) |  |
| **Inpatient** | 0/614 (0) | 0/614 (0) | 0/614 (0) | 0/614 (0) | 0/614 (0) | 0/614 (0) | 0/614 (0) | 0/614 (0) | 0/614 (0) | 0/614 (0) | 0/614 (0) | 0/614 (0) | 0/614 (0) | 0/614 (0) | 0/614 (0) | 0/614 (0) | 0/614 (0) |  |
| **Mortality** | 0/614 (0) | 0/614 (0) | 0/614 (0) | 0/614 (0) | 0/614 (0) | 0/614 (0) | 0/614 (0) | 0/614 (0) | 0/614 (0) | 0/614 (0) | 0/614 (0) | 0/614 (0) | 0/614 (0) | 0/614 (0) | 0/614 (0) | 0/614 (0) | 0/614 (0) |  |
| **Other** | 0/614 (0) | 0/614 (0) | 0/614 (0) | 0/614 (0) | 0/614 (0) | 0/614 (0) | 0/614 (0) | 0/614 (0) | 1/614 (0.16) | 0/614 (0) | 0/614 (0) | 0/614 (0) | 0/614 (0) | 0/614 (0) | 0/614 (0) | 0/614 (0) | 1/614 (0.16) |  |

Table S16. Pulmonary post-vaccination symptoms in healthy children

|  | | **Cough** | **Irregular breathing** | **Rapid breathing** | **Shortness of breath on exertion** | **Shortness of breath at rest** | **Other complaints** |
| --- | --- | --- | --- | --- | --- | --- | --- |
| **After First vaccination** | | 1/396 (0.25) | 0/396 (0) | 0/396 (0) | 1/396 (0.25) | 0/396 (0) | 0/396 (0) |
| **After Second vaccination** | | 2/218 (0.92) | 0/218 (0) | 0/218 (0) | 0/218 (0) | 0/218 (0) | 0/218 (0) |
| **Beginning & Duration** | |  |  |  |  |  |  |
| **Beginning (days after vaccination, mean ±SD/N)** | 3±1 /3 | 0±0/ 0 | 0±0/ 0 | 2± 0/ 1 | 0±0/ 0 | 0±0/ 0 |  |
| **Duration (days, mean ±SD/N)** | 3.67± 2.31/ 3 | 0±0/ 0 | 0±0/ 0 | 1± 0/ 1 | 0±0/ 0 | 0±0/ 0 |  |
| **Aftermath** | |  |  |  |  |  |  |
| **Oral medication (e.g. cough syrup)** | 0/614 (0) | 0/614 (0) | 0/614 (0) | 0/614 (0) | 0/614 (0) | 0/614 (0) |  |
| **Inhalative medication e.g. salbutamol** | 0/614 (0) | 0/614 (0) | 0/614 (0) | 0/614 (0) | 0/614 (0) | 0/614 (0) |  |
| **Ambulatory** | 0/614 (0) | 0/614 (0) | 0/614 (0) | 0/614 (0) | 0/614 (0) | 0/614 (0) |  |
| **Innpatient** | 0/614 (0) | 0/614 (0) | 0/614 (0) | 0/614 (0) | 0/614 (0) | 0/614 (0) |  |
| **Mortality** | 0/614 (0) | 0/614 (0) | 0/614 (0) | 0/614 (0) | 0/614 (0) | 0/614 (0) |  |
| **Other** | 0/614 (0) | 0/614 (0) | 0/614 (0) | 0/614 (0) | 0/614 (0) | 0/614 (0) |  |

Table S17. Cardiovascular post-vaccination symptoms in healthy children

|  | | **Tachycardia** | **Chest pain** | **Chest tightness** | **Cold hands and feet** | **Discoloration of the hands and feet** | **Other discomforts** |
| --- | --- | --- | --- | --- | --- | --- | --- |
| **After First vaccination** | | 1/396 (0.25) | 0/396 (0) | 0/396 (0) | 0/396 (0) | 0/396 (0) | 2/396 (0.51) |
| **After Second vaccination** | | 0/218 (0) | 0/218 (0) | 0/218 (0) | 0/218 (0) | 0/218 (0) | 0/218 (0) |
| **Beginning & Duration** | |  |  |  |  |  |  |
| **Beginning (days after vaccination, mean ±SD/N)** | 0± 0/ 1 | 0±0/ 0 | 0±0/ 0 | 0±0/ 0 | 0±0/ 0 | 0± 0/ 2 |  |
| **Duration (days, mean ±SD/N)** | 2± 0/ 1 | 0±0/ 0 | 0±0/ 0 | 0±0/ 0 | 0±0/ 0 | 0.5± 0/ 2 |  |
| **Aftermath** | |  |  |  |  |  |  |
| **Pain Medication** |  |  | 0/614 (0) |  |  | 0/614 (0) |  |
| **Ambulatory** | 0/614 (0) | 0/614 (0) | 0/614 (0) | 0/614 (0) | 1/614 (0.16) | 0/614 (0) |  |
| **Inpatient** | 0/614 (0) | 0/614 (0) | 0/614 (0) | 0/614 (0) | 1/614 (0.16) | 0/614 (0) |  |
| **Mortality** | 0/614 (0) | 0/614 (0) | 0/614 (0) | 0/614 (0) | 0/614 (0) | 0/614 (0) |  |
| **Other** | 0/614 (0) | 0/614 (0) | 0/614 (0) | 0/614 (0) | 0/614 (0) | 0/614 (0) |  |

Table S18. Neurological post-vaccination symptoms in healthy children

|  | | **Headache** | **Circulatory collapse** | **Dizziness** | **Sensory disturbances** | **Locomotor disturbances** | **Loss of consciousness** | **Neuralgia** | **Seizure** | **Paralysis of facial muscles** | **Other complaints** |
| --- | --- | --- | --- | --- | --- | --- | --- | --- | --- | --- | --- |
| **After First vaccination** | | 33/396 (8.33) | 0/396 (0) | 6/396 (1.52) | 1/396 (0.25) | 0/396 (0) | 0/396 (0) | 0/396 (0) | 0/396 (0) | 0/396 (0) | 0/396 (0) |
| **After Second vaccination** | | 19/218 (8.72) | 0/218 (0) | 2/218 (0.92) | 0/218 (0) | 0/218 (0) | 0/218 (0) | 0/218 (0) | 0/218 (0) | 0/218 (0) | 0/218 (0) |
| **Beginning & Duration** | |  |  |  |  |  |  |  |  |  |  |
| **Beginning (days after vaccination, mean ±SD/N)** | 1.15± 2.37/ 52 | 0±0/ 0 | 3± 3.30/ 8 | 0± 0/ 1 | 0±0/ 0 | 0±0/ 0 | 0±0/ 0 | 0±0/ 0 | 0±0/ 0 | 0±0/ 0 |  |
| **Duration (days, mean ±SD/N)** | 1.35± 0.81/ 51 | 0±0/ 0 | 1.9± 1.16/ 8 | 0.5± 0/ 1 | 0±0/ 0 | 0±0/ 0 | 0±0/ 0 | 0±0/ 0 | 0±0/ 0 | 0±0/ 0 |  |
| **Aftermath** | |  |  |  |  |  |  |  |  |  |  |
| **Pain Medication** | 18/614 (2.93) |  |  |  |  |  | 0/614 (0) |  |  | 0/614 (0) |  |
| **Ambulatory** | 0/614 (0) | 0/614 (0) | 1/614 (0.16) | 0/614 (0) | 0/614 (0) | 0/614 (0) | 0/614 (0) | 0/614 (0) | 0/614 (0) | 0/614 (0) |  |
| **Inpatient** | 0/614 (0) | 0/614 (0) | 0/614 (0) | 0/614 (0) | 0/614 (0) | 0/614 (0) | 0/614 (0) | 0/614 (0) | 0/614 (0) | 0/614 (0) |  |
| **Mortality** | 0/614 (0) | 0/614 (0) | 0/614 (0) | 0/614 (0) | 0/614 (0) | 0/614 (0) | 0/614 (0) | 0/614 (0) | 0/614 (0) | 0/614 (0) |  |
| **Other** | 0/614 (0) | 0/614 (0) | 0/614 (0) | 0/614 (0) | 0/614 (0) | 0/614 (0) | 0/614 (0) | 0/614 (0) | 0/614 (0) | 0/614 (0) |  |

Table S19. Psychological post-vaccination symptoms in healthy children

|  | | **Concentration problems** | **Memory disorder** | **Sleeping disorder** | **Aggressive behavior** | **Nervousness** | **Hyperactivity** | **Anxiety** | **Sadness/ depression** | **Mood swings** | **Other complaints** |
| --- | --- | --- | --- | --- | --- | --- | --- | --- | --- | --- | --- |
| **After First vaccination** | | 2/396 (0.51) | 0/396 (0) | 1/396 (0.25) | 0/396 (0) | 1/396 (0.25) | 3/396 (0.76) | 0/396 (0) | 1/396 (0.25) | 2/396 (0.51) | 1/396 (0.25) |
| **After Second vaccination** | | 0/218 (0) | 0/218 (0) | 0/218 (0) | 0/218 (0) | 0/218 (0) | 0/218 (0) | 0/218 (0) | 0/218 (0) | 2/218 (0.92) | 0/218 (0) |
| **Beginning & Duration** | |  |  |  |  |  |  |  |  |  |  |
| **Beginning (days after vaccination, mean±SD/N)** | 1± 0/ 2 | 0±0/ 0 | 1± 0/ 1 | 0±0/ 0 | 0± 0/ 1 | 0.33± 0.58/ 3 | 0±0/ 0 | 4± 0/ 1 | 2.25± 3.2/ 4 | 2± 0/ 1 |  |
| **Duration (days, mean±SD/N)** | 8.5± 2.12/ 2 | 0±0/ 0 | 10± 0/ 1 | 0±0/ 0 | 10± 0/ 1 | 5.83± 4.86/ 3 | 0±0/ 0 | 2± 0/ 1 | 6.25± 6.4/ 4 | 2± 0/ 1 |  |
| **Aftermath** | |  |  |  |  |  |  |  |  |  |  |
| **Ambulatory** | 1/614 (0.16) | 0/614 (0) | 1/614 (0.16) | 0/614 (0) | 1/614 (0.16) | 1/614 (0.16) | 0/614 (0) | 0/614 (0) | 0/614 (0) | 0/614 (0) |  |
| **Inpatient** | 0/614 (0) | 0/614 (0) | 0/614 (0) | 0/614 (0) | 0/614 (0) | 0/614 (0) | 0/614 (0) | 0/614 (0) | 0/614 (0) | 0/614 (0) |  |
| **Mortality** | 0/614 (0) | 0/614 (0) | 0/614 (0) | 0/614 (0) | 0/614 (0) | 0/614 (0) | 0/614 (0) | 0/614 (0) | 0/614 (0) | 0/614 (0) |  |
| **Other** | 0/614 (0) | 0/614 (0) | 0/614 (0) | 0/614 (0) | 0/614 (0) | 0/614 (0) | 0/614 (0) | 0/614 (0) | 1/614 (0.16) | 0/614 (0) |  |

Table S20. Dermatological post-vaccination symptoms in healthy children

|  | | **Local rash** | **Rash all over the body** | **Swelling of lymph nodes** | **Painful lymph nodes** | **Extensive redness of the skin** | **Other skin discolorations** | **Wheals/ hives** | **Blisters/ pustules** | **Petechiae** | **Hematoma** | **Dry skin** | **Eczema** | **Open skin areas/ wounds** | **Pruritus** | **Other complaints** |
| --- | --- | --- | --- | --- | --- | --- | --- | --- | --- | --- | --- | --- | --- | --- | --- | --- |
| **After First vaccination** | | 0/396 (0) | 0/396 (0) | 7/396 (1.77) | 6/396 (1.52) | 0/396 (0) | 0/396 (0) | 0/396 (0) | 0/396 (0) | 0/396 (0) | 0/396 (0) | 0/396 (0) | 1/396 (0.25) | 0/396 (0) | 1/396 (0.25) | 0/396 (0) |
| **After Second vaccination** | | 4/218 (1.83) | 0/218 (0) | 5/218 (2.29) | 6/218 (2.75) | 0/218 (0) | 0/218 (0) | 1/218 (0.46) | 0/218 (0) | 0/218 (0) | 0/218 (0) | 2/218 (0.92) | 0/218 (0) | 0/218 (0) | 1/218 (0.46) | 0/218 (0) |
| **Beginning & Duration** | |  |  |  |  |  |  |  |  |  |  |  |  |  |  |  |
| **Beginning (days after vaccination, mean±SD/N)** | 4.25±4.65/ 4 | 0±0/ 0 | 1.42± 0.51/ 12 | 1.42± 0.67/ 12 | 0±0/ 0 | 0±0/ 0 | 6±0 /1 | 0±0/ 0 | 0±0/ 0 | 0±0/ 0 | 5± 1.41/ 2 | 3±0/ 1 | 0±0/ 0 | 3.5± 3.54/ 2 | 0±0/ 0 |  |
| **Duration (days, mean±SD/N)** | 2.13±1.44/ 4 | 0±0/ 0 | 4.17± 2.86/ 12 | 3.5± 2.61/ 12 | 0±0/ 0 | 0±0/ 0 | 0.5± 0/ 1 | 0±0/ 0 | 0±0/ 0 | 0±0/ 0 | 14± 0/ 2 | 1±0/ 1 | 0±0/ 0 | 0.5± 0/ 2 | 0±0/ 0 |  |
| **Aftermath** | |  |  |  |  |  |  |  |  |  |  |  |  |  |  |  |
| **Allergy medication/ pain medication** | 0/614 (0) | 0/614 (0) | 1/614 (0.16) | 2/614 (0.33) | 0/614 (0) | 0/614 (0) | 0/614 (0) | 0/614 (0) | 0/614 (0) | 0/614 (0) | 0/614 (0) | 0/614 (0) | 0/614 (0) | 0/614 (0) | 0/614 (0) |  |
| **Topical cremes** | 1/614 (0.16) | 0/614 (0) | 0/614 (0) | 0/614 (0) | 0/614 (0) | 0/614 (0) | 1/614 (0.16) | 0/614 (0) | 0/614 (0) | 0/614 (0) | 0/614 (0) | 1/614 (0.16) | 0/614 (0) | 1/614 (0.16) | 0/614 (0) |  |
| **Ambulatory** | 0/614 (0) | 0/614 (0) | 1/614 (0.16) | 0/614 (0) | 0/614 (0) | 0/614 (0) | 0/614 (0) | 0/614 (0) | 0/614 (0) | 0/614 (0) | 0/614 (0) | 0/614 (0) | 0/614 (0) | 0/614 (0) | 0/614 (0) |  |
| **Inpatient** | 0/614 (0) | 0/614 (0) | 0/614 (0) | 0/614 (0) | 0/614 (0) | 0/614 (0) | 0/614 (0) | 0/614 (0) | 0/614 (0) | 0/614 (0) | 0/614 (0) | 0/614 (0) | 0/614 (0) | 0/614 (0) | 0/614 (0) |  |
| **Mortality** | 0/614 (0) | 0/614 (0) | 0/614 (0) | 0/614 (0) | 0/614 (0) | 0/614 (0) | 0/614 (0) | 0/614 (0) | 0/614 (0) | 0/614 (0) | 0/614 (0) | 0/614 (0) | 0/614 (0) | 0/614 (0) | 0/614 (0) |  |
| **Other** | 0/614 (0) | 0/614 (0) | 0/614 (0) | 0/614 (0) | 0/614 (0) | 0/614 (0) | 0/614 (0) | 0/614 (0) | 0/614 (0) | 0/614 (0) | 1/614 (0.16) | 0/614 (0) | 0/614 (0) | 0/614 (0) | 0/614 (0) |  |

## **Post-vaccination symptoms with comorbidities**

Table S21. Local post-vaccination symptoms in children with comorbidities

|  | | **Redness at the injection site** | **Swelling at the injection site** | **Pain at the injection site** | **Other local discomfort** |
| --- | --- | --- | --- | --- | --- |
| **After First vaccination** | | 9/110 (8.18) | 11/110 (10.00) | 67/110 (60.91) | 2/110 (1.82) |
| **After Second vaccination** | | 9/69 (13.04) | 12/69 (17.39) | 42/69 (60.87) | 2/69 (2.90) |
| **Beginning & Duration** | |  |  |  |  |
| **Beginning (days after vaccination, mean ± SD/N)** | 0.83±1.19/ 18 | 0.78±1.06/ 23 | 0.57±0.46/ 109 | 1.88±2.10/ 4 |  |
| **Duration (days, mean ± SD/N)** | 2.28±1.13/ 18 | 2.26±1.42/ 23 | 2.04±1.17/ 109 | 1.50±1.29/ 4 |  |
| **Aftermath** | |  |  |  |  |
| **Pain Medication** |  |  | 3/179 (1.68) |  |  |
| **Ambulatory** | 0/179 (0) | 0/179 (0) | 0/179 (0) | 0/179 (0) |  |
| **Inpatient** | 0/179 (0) | 0/179 (0) | 0/179 (0) | 0/179 (0) |  |
| **Mortality** | 0/179 (0) | 0/179 (0) | 0/179 (0) | 0/179 (0) |  |
| **Other** | 0/179 (0) | 1/179 (0.56) | 2/179 (1.12) | 0/179 (0) |  |

Table S22. General post-vaccination symptoms in children with comorbidities

|  | | **Fever**  **(Body Temperature**  **≥ 38,5°C)** | **Chills** | **Hot flashes** | **Fatigue** | **Flu- like symptoms** | **Generalized weakness** | **Malaise** | **MIS-C** | **Other general complaints** |
| --- | --- | --- | --- | --- | --- | --- | --- | --- | --- | --- |
| **After First vaccination** | | 5/110 (4.55) | 2/110 (1.82) | 0/110 (0) | 19/110 (17.27) | 4/110 (3.64) | 8/110 (7.27) | 3/110 (2.73) | 0/110 (0) | 3/110 (2.73) |
| **After Second vaccination** | | 3/69 (4.35) | 1/69 (1.45) | 0/69 (0) | 17/69 (24.64) | 1/69 (1.45) | 5/69 (7.25) | 5/69 (7.25) | 0/69 (0) | 2/69 (2.90) |
| **Beginning & Duration** | |  |  |  |  |  |  |  |  |  |
| **Beginning (days after vaccination, mean ± SD/N)** | 1.63±0.74/8 | 1±0/ 3 | 0±0/ 0 | 0.79±0.59/ 36 | 1.2±0.45/ 5 | 0.85±0.32/ 13 | 1.5±1.07/ 8 | 0±0/ 0 | 1.3±1.2/ 5 |  |
| **Duration (days, mean ± SD/N)** | 2±1.31/ 8 | 1±1/ 3 | 0±0/ 0 | 2±1.72/ 36 | 6.4±5.59/ 5 | 2.31±2.75/ 13 | 4.71±4.72/ 8 | 0±0/ 0 | 4.8±7.12/ 5 |  |
| **Aftermath** | |  |  |  |  |  |  |  |  |  |
| **Analgesics/Pain medication** | 5/179 (2.79) | 1/179 (0.56) | 0/179 (0) |  | 3/179 (1.68) |  | 3/179 (1.68) | 0/179 (0) | 3/179 (1.68) |  |
| **Ambulatory** | 1/179 (0.56) | 0/179 (0) | 0/179 (0) | 0/179 (0) | 0/179 (0) | 0/179 (0) | 0/179 (0) | 0/179 (0) | 0/179 (0) |  |
| **Inpatient** | 0/179 (0) | 0/179 (0) | 0/179 (0) | 0/179 (0) | 0/179 (0) | 0/179 (0) | 0/179 (0) | 0/179 (0) | 0/179 (0) |  |
| **Mortality** | 0/179 (0) | 0/179 (0) | 0/179 (0) | 0/179 (0) | 0/179 (0) | 0/179 (0) | 0/179 (0) | 0/179 (0) | 0/179 (0) |  |
| **Other** | 0/179 (0) | 0/179 (0) | 0/179 (0) | 0/179 (0) | 0/179 (0) | 0/179 (0) | 0/179 (0) | 0/179 (0) | 0/179 (0) |  |

Table S23. Musculoskeletal post-vaccination symptoms in children with comorbidities

|  | | **Muscle weakness** | **Muscle Pain** | **Muscle twitching** | **Neck/back pain** | **Pain in the arms** | **Pain in the legs** | **Joint pain** | **Joint swelling** | **Pain in limbs** | **Other complaints** |
| --- | --- | --- | --- | --- | --- | --- | --- | --- | --- | --- | --- |
| **After First vaccination** | | 1/110 (0.91) | 1/110 (0.91) | 0/110 (0) | 0/110 (0) | 8/110 (7.27) | 1/110 (0.91) | 2/110 (1.82) | 0/110 (0) | 2/110 (1.82) | 1/110 (0.91) |
| **After Second vaccination** | | 0/69 (0) | 1/69 (1.45) | 0/69 (0) | 0/69 (0) | 3/69 (4.35) | 0/69 (0) | 2/69 (2.90) | 0/69 (0) | 0/69 (0) | 0/69 (0) |
| **Beginning & Duration** | |  |  |  |  |  |  |  |  |  |  |
| **Beginning (days after vaccination, mean ± SD/N)** | 1±0/1 | 1.5±0.58/ 2 | 0±0/ 0 | 0±0/ 0 | 0.75±0.38/ 11 | 1±0/ 1 | 4.75±2.87/ 4 | 0±0/ 0 | 1±0/ 2 | 1±0/ 1 |  |
| **Duration (days, mean ± SD/N)** | 3±0/ 1 | 4.5±1.53/ 2 | 0±0/ 0 | 0±0/ 0 | 2.64±1.75/ 11 | 3±0/ 1 | 3.33±4.16/3 | 0±0/ 0 | 3.5±0.71/ 2 | 1±0/ 1 |  |
| **Aftermath** | |  |  |  |  |  |  |  |  |  |  |
| **Pain medication** |  | 0/179 (0) |  | 0/179 (0) | 0/179 (0) | 1/179 (0.56) | 0/179 (0) | 0/179 (0) | 0/179 (0) | 0/179 (0) |  |
| **Ambulatory** | 0/179 (0) | 0/179 (0) | 0/179 (0) | 0/179 (0) | 0/179 (0) | 0/179 (0) | 0/179 (0) | 0/179 (0) | 0/179 (0) | 0/179 (0) |  |
| **Inpatient** | 0/179 (0) | 0/179 (0) | 0/179 (0) | 0/179 (0) | 0/179 (0) | 0/179 (0) | 0/179 (0) | 0/179 (0) | 0/179 (0) | 0/179 (0) |  |
| **Mortality** | 0/179 (0) | 0/179 (0) | 0/179 (0) | 0/179 (0) | 0/179 (0) | 0/179 (0) | 0/179 (0) | 0/179 (0) | 0/179 (0) | 0/179 (0) |  |
| **Other** | 0/179 (0) | 0/179 (0) | 0/179 (0) | 0/179 (0) | 0/179 (0) | 0/179 (0) | 0/179 (0) | 0/179 (0) | 0/179 (0) | 0/179 (0) |  |

Table S24. Gastrointestinal post-vaccination symptoms in children with comorbidities

|  | | **Pain in upper abdomen** | **Pain in lower abdomen** | **Nausea/vomiting** | **Obstipation** | **Diarrhea** | **Other stool changes** | **Unwanted weight changes** | **Other complaints** |
| --- | --- | --- | --- | --- | --- | --- | --- | --- | --- |
| **After First vaccination** | | 1/110 (0.91) | 3/110 (2.73) | 7/110 (6.36) | 0/110 (0) | 1/110 (0.91) | 0/110 (0) | 0/110 (0) | 0/110 (0) |
| **After Second vaccination** | | 2/69 (2.90) | 3/69 (4.35) | 4/69 (5.80) | 0/69 (0) | 0/69 (0) | 1/69 (1.45) | 0/69 (0) | 0/69 (0) |
| **Beginning & Duration** | |  |  |  |  |  |  |  |  |
| **Beginning (days after vaccination, mean ± SD/N)** | 2.67±1.53/ 3 | 3.33±2.25/ 6 | 1.77± 2.02/ 11 | 0±0/ 0 | 2±0/ 1 | 3± 0/ 1 | 0±0/ 0 | 0±0/ 0 |  |
| **Duration (days, mean ± SD/N)** | 6±9.54/ 3 | 6.83±5.56/ 6 | 2.64± 4.95/ 11 | 0±0/ 0 | 2±0/ 1 | 10± 0/ 1 | 0±0/ 0 | 0±0/ 0 |  |
| **Aftermath** | |  |  |  |  |  |  |  |  |
| **Pain Medication** | 1/179 (0.56) | 2/179 (1.12) | 3/179 (1.68) | 0/179 (0) | 0/179 (0) | 0/179 (0) |  |  |  |
| **Antiemetics** | 1/179 (0.56) | 1/179 (0.56) | 1/179 (0.56) | 0/179 (0) | 0/179 (0) | 0/179 (0) | 0/179 (0) | 0/179 (0) |  |
| **Ambulatory** | 1/179 (0.56) | 1/179 (0.56) | 0/179 (0) | 0/179 (0) | 0/179 (0) | 0/179 (0) | 0/179 (0) | 0/179 (0) |  |
| **Inpatient** | 0/179 (0) | 0/179 (0) | 0/179 (0) | 0/179 (0) | 0/179 (0) | 0/179 (0) | 0/179 (0) | 0/179 (0) |  |
| **Mortality** | 0/179 (0) | 0/179 (0) | 0/179 (0) | 0/179 (0) | 0/179 (0) | 0/179 (0) | 0/179 (0) | 0/179 (0) |  |
| **Other** | 0/179 (0) | 0/179 (0) | 0/179 (0) | 0/179 (0) | 0/179 (0) | 0/179 (0) | 0/179 (0) | 0/179 (0) |  |

Table S25. Otolaryngologic post-vaccination symptoms in children with comorbidities

|  | | **Nose-bleed** | **Redness of the oral mucosa** | **Swelling of the tongue** | **Swelling of the lips** | **Discomfort in the mouth** | **Toothache** | **Bleeding of the gums** | **Tightness in the throat** | **Sore throat** | **Earache** | **Hoarse-ness** | **Facial swelling** | **Swollen lymph nodes** | **Painful lymph nodes** | **Olfactory disorder** | **Taste disorder** | **Other complaints** |
| --- | --- | --- | --- | --- | --- | --- | --- | --- | --- | --- | --- | --- | --- | --- | --- | --- | --- | --- |
| **After First vaccination** | | 0/110 (0) | 0/110 (0) | 0/110 (0) | 0/110 (0) | 0/110 (0) | 1/110 (0.91) | 0/110 (0) | 0/110 (0) | 1/110 (0.91) | 0/110 (0) | 0/110 (0) | 1/110 (0.91) | 0/110 (0) | 0/110 (0) | 0/110 (0) | 0/110 (0) | 2/110 (1.82) |
| **After Second vaccination** | | 0/69 (0) | 0/69 (0) | 0/69 (0) | 0/69 (0) | 0/69 (0) | 0/69 (0) | 0/69 (0) | 0/69 (0) | 2/69 (2.90) | 0/69 (0) | 1/69 (1.45) | 0/69 (0) | 0/69 (0) | 0/69 (0) | 0/69 (0) | 0/69 (0) | 1/69 (1.45) |
| **Beginning & Duration** | |  |  |  |  |  |  |  |  |  |  |  |  |  |  |  |  |  |
| **Beginning (days after vaccination, mean ±SD/N)** | 0±0/ 0 | 0±0/ 0 | 0±0/ 0 | 0±0/ 0 | 0±0/ 0 | 3±0/ 1 | 0±0/ 0 | 0±0/ 0 | 2.67±1.53 /3 | 0±0/ 0 | 14± 0/ 1 | 5± 0/ 1 | 0±0/ 0 | 0±0/ 0 | 0±0/ 0 | 0±0/ 0 | 2.33± 0.58/ 3 |  |
| **Duration (days, mean ±SD/N)** | 0±0/ 0 | 0±0/ 0 | 0±0/ 0 | 0±0/ 0 | 0±0/ 0 | 1±0/ 1 | 0±0/ 0 | 0±0/ 0 | 6.67±2.52 /3 | 0±0/ 0 | 3± 0/ 1 | 8± 0/ 1 | 0±0/ 0 | 0±0/ 0 | 0±0/ 0 | 0±0/ 0 | 3.33± 0.58/ 3 |  |
| **Aftermath** | |  |  |  |  |  |  |  |  |  |  |  |  |  |  |  |  |  |
| **Pain Medication** |  |  |  |  |  | 0/179 (0) | 0/179 (0) | 0/179 (0) | 0/179 (0) | 0/179 (0) | 1/179 (0.56) |  | 0/179 (0) | 0/179 (0) |  |  | 0/179 (0) |  |
| **Ambulatory** | 0/179 (0) | 0/179 (0) | 0/179 (0) | 0/179 (0) | 0/179 (0) | 0/179 (0) | 0/179 (0) | 0/179 (0) | 0/179 (0) | 0/179 (0) | 0/179 (0) | 0/179 (0) | 0/179 (0) | 0/179 (0) | 0/179 (0) | 0/179 (0) | 0/179 (0) |  |
| **Inpatient** | 0/179 (0) | 0/179 (0) | 0/179 (0) | 0/179 (0) | 0/179 (0) | 0/179 (0) | 0/179 (0) | 0/179 (0) | 0/179 (0) | 0/179 (0) | 0/179 (0) | 0/179 (0) | 0/179 (0) | 0/179 (0) | 0/179 (0) | 0/179 (0) | 0/179 (0) |  |
| **Mortality** | 0/179 (0) | 0/179 (0) | 0/179 (0) | 0/179 (0) | 0/179 (0) | 0/179 (0) | 0/179 (0) | 0/179 (0) | 0/179 (0) | 0/179 (0) | 0/179 (0) | 0/179 (0) | 0/179 (0) | 0/179 (0) | 0/179 (0) | 0/179 (0) | 0/179 (0) |  |
| **Other** | 0/179 (0) | 0/179 (0) | 0/179 (0) | 0/179 (0) | 0/179 (0) | 0/179 (0) | 0/179 (0) | 0/179 (0) | 0/179 (0) | 0/179 (0) | 0/179 (0) | 0/179 (0) | 0/179 (0) | 0/179 (0) | 0/179 (0) | 0/179 (0) | 1/179 (0.56) |  |

Table S26. Pulmonary post-vaccination symptoms in children with comorbidities

|  | | **Cough** | **Irregular breathing** | **Rapid breathing** | **Shortness of breath on exertion** | **Shortness of breath at rest** | **Other complaints** |
| --- | --- | --- | --- | --- | --- | --- | --- |
| **After First vaccination** | | 3/110 (2.73) | 0/110 (0) | 1/110 (0.91) | 1/110 (0.91) | 0/110 (0) | 1/110 (0.91) |
| **After Second vaccination** | | 1/69 (1.45) | 0/69 (0) | 0/69 (0) | 0/69 (0) | 0/69 (0) | 3/69 (4.35) |
| **Beginning & Duration** | |  |  |  |  |  |  |
| **Beginning (days after vaccination, mean ±SD/N)** | 4.75±6.24 /4 | 0±0/ 0 | 4± 0/ 1 | 2± 0/ 1 | 0±0/ 0 | 4.5±6.35/ 4 |  |
| **Duration (days, mean ±SD/N)** | 5.75± 5.91/ 4 | 0±0/ 0 | 2± 0/ 1 | 10± 0/ 1 | 0±0/ 0 | 3.5±1.73/ 4 |  |
| **Aftermath** | |  |  |  |  |  |  |
| **Oral medication (e.g. cough syrup)** | 1/179 (0.56) | 0/179 (0) | 0/179 (0) | 1/179 (0.56) | 0/179 (0) | 0/179 (0) |  |
| **Inhalative medication e.g. salbutamol** | 1/179 (0.56) | 0/179 (0) | 1/179 (0.56) | 1/179 (0.56) | 0/179 (0) | 1/179 (0.56) |  |
| **Ambulatory** | 0/179 (0) | 0/179 (0) | 0/179 (0) | 0/179 (0) | 0/179 (0) | 0/179 (0) |  |
| **Inpatient** | 0/179 (0) | 0/179 (0) | 0/179 (0) | 0/179 (0) | 0/179 (0) | 0/179 (0) |  |
| **Mortality** | 0/179 (0) | 0/179 (0) | 0/179 (0) | 0/179 (0) | 0/179 (0) | 0/179 (0) |  |
| **Other** | 2/179 (1.12) | 0/179 (0) | 0/179 (0) | 0/179 (0) | 0/179 (0) | 2/179 (1.12) |  |

Table S27. Cardiovascular post-vaccination symptoms in children with comorbidities

|  | | **Tachycardia** | **Chest pain** | **Chest tightness** | **Cold hands and feet** | **Discoloration of the hands and feet** | **Other discomforts** |
| --- | --- | --- | --- | --- | --- | --- | --- |
| **After First vaccination** | | 0/110 (0) | 1/110 (0.91) | 0/110 (0) | 0/110 (0) | 0/110 (0) | 1/110 (0.91) |
| **After Second vaccination** | | 0/69 (0) | 0/69 (0) | 0/69 (0) | 0/69 (0) | 0/69 (0) | 0/69 (0) |
| **Beginning & Duration** | |  |  |  |  |  |  |
| **Beginning (days after vaccination, mean ±SD/N)** | 0±0/ 0 | 0.5± 0/ 1 | 0±0/ 0 | 0±0/ 0 | 0±0/ 0 | 1± 0/ 1 |  |
| **Duration (days, mean ±SD/N)** | 0±0/ 0 | 0± 0/ 1 | 0±0/ 0 | 0±0/ 0 | 0±0/ 0 | 3± 0/ 1 |  |
| **Aftermath** | |  |  |  |  |  |  |
| **Pain Medication** |  |  | 0/179 (0) |  |  | 0/179 (0) |  |
| **Ambulatory** | 0/179 (0) | 0/179 (0) | 0/179 (0) | 0/179 (0) | 0/179 (0) | 0/179 (0) |  |
| **Inpatient** | 0/179 (0) | 0/179 (0) | 0/179 (0) | 0/179 (0) | 0/179 (0) | 0/179 (0) |  |
| **Mortality** | 0/179 (0) | 0/179 (0) | 0/179 (0) | 0/179 (0) | 0/179 (0) | 0/179 (0) |  |
| **Other** | 0/179 (0) | 0/179 (0) | 0/179 (0) | 0/179 (0) | 0/179 (0) | 0/179 (0) |  |

Table S28. Neurological post-vaccination symptoms in children with comorbidities

|  | | **Headache** | **Circulatory collapse** | **Dizziness** | **Sensory disturbances** | **Locomotor disturbances** | **Loss of consciousness** | **Neuralgia** | **Seizure** | **Paralysis of facial muscles** | **Other complaints** |
| --- | --- | --- | --- | --- | --- | --- | --- | --- | --- | --- | --- |
| **After First vaccination** | | 16/110 (14.55) | 0/110 (0) | 3/110 (2.73) | 1/110 (0.91) | 0/110 (0) | 0/110 (0) | 0/110 (0) | 0/110 (0) | 0/110 (0) | 0/110 (0) |
| **After Second vaccination** | | 8/69 (11.43) | 0/69 (0) | 1/69 (1.43) | 0/69 (0) | 0/69 (0) | 0/69 (0) | 0/69 (0) | 0/69 (0) | 0/69 (0) | 0/69 (0) |
| **Beginning & Duration** | |  |  |  |  |  |  |  |  |  |  |
| **Beginning (days after vaccination, mean ±SD/N)** | 1.20± 0,85/ 23 | 0±0/ 0 | 1.38± 1.11/ 4 | 0.5± 0/ 1 | 0±0/ 0 | 0±0/ 0 | 0±0/ 0 | 0±0/ 0 | 0±0/ 0 | 0±0/ 0 |  |
| **Duration (days, mean ±SD/N)** | 1.27± 1.49/ 22 | 0±0/ 0 | 0.25± 0.5/ 4 | 0± 0/ 1 | 0±0/ 0 | 0±0/ 0 | 0±0/ 0 | 0±0/ 0 | 0±0/ 0 | 0±0/ 0 |  |
| **Aftermath** | |  |  |  |  |  |  |  |  |  |  |
| **Pain Medication** | 8/179 (4.47) |  |  |  |  |  | 0/179 (0) |  |  | 0/179 (0) |  |
| **Ambulatory** | 0/179 (0) | 0/179 (0) | 0/179 (0) | 0/179 (0) | 0/179 (0) | 0/179 (0) | 0/179 (0) | 0/179 (0) | 0/179 (0) | 0/179 (0) |  |
| **Inpatient** | 0/179 (0) | 0/179 (0) | 0/179 (0) | 0/179 (0) | 0/179 (0) | 0/179 (0) | 0/179 (0) | 0/179 (0) | 0/179 (0) | 0/179 (0) |  |
| **Mortality** | 0/179 (0) | 0/179 (0) | 0/179 (0) | 0/179 (0) | 0/179 (0) | 0/179 (0) | 0/179 (0) | 0/179 (0) | 0/179 (0) | 0/179 (0) |  |
| **Other** | 0/179 (0) | 0/179 (0) | 0/179 (0) | 0/179 (0) | 0/179 (0) | 0/179 (0) | 0/179 (0) | 0/179 (0) | 0/179 (0) | 0/179 (0) |  |

Table S29. Psychological post-vaccination symptoms in children with comorbidities

|  | | **Concentration problems** | **Memory disorder** | **Sleeping disorder** | **Aggressive behavior** | **Nervousness** | **Hyperactivity** | **Anxiety** | **Sadness/ depression** | **Mood swings** | **Other complaints** |
| --- | --- | --- | --- | --- | --- | --- | --- | --- | --- | --- | --- |
| **After First vaccination** | | 0/110 (0) | 0/110 (0) | 3/110 (2.73) | 0/110 (0) | 0/110 (0) | 1/110 (0.91) | 0/110 (0) | 0/110 (0) | 1/110 (0.91) | 0/110 (0) |
| **After Second vaccination** | | 2/69 (2.90) | 0/69 (0) | 4/69 (5.80) | 0/69 (0) | 0/69 (0) | 0/69 (0) | 2/69 (2.90) | 1/69 (1.45) | 1/69 (1.45) | 0/69 (0) |
| **Beginning & Duration** | |  |  |  |  |  |  |  |  |  |  |
| **Beginning (days after vaccination,mean±SD/N)** | 2± 1.41/ 2 | 0±0/ 0 | 2.79± 3.49/ 7 | 0±0/ 0 | 0±0/ 0 | 2± 0/ 1 | 0.75± 0.35/ 2 | 0.5± 0/ 1 | 1.5± 0.71/ 2 | 0±0/ 0 |  |
| **Duration (days, mean±SD/N)** | 6± 5.66/ 2 | 0±0/ 0 | 8.14± 8.47/ 7 | 0±0/ 0 | 0±0/ 0 | 4± 0/ 1 | 9± 11.31/ 2 | 17± 0/ 1 | 9± 11.31/ 2 | 0±0/ 0 |  |
| **Aftermath** | |  |  |  |  |  |  |  |  |  |  |
| **Ambulatory** | 0/179 (0) | 0/179 (0) | 0/179 (0) | 0/179 (0) | 0/179 (0) | 0/179 (0) | 0/179 (0) | 0/179 (0) | 0/179 (0) | 0/179 (0) |  |
| **Inpatient** | 0/179 (0) | 0/179 (0) | 0/179 (0) | 0/179 (0) | 0/179 (0) | 0/179 (0) | 0/179 (0) | 0/179 (0) | 0/179 (0) | 0/179 (0) |  |
| **Mortality** | 0/179 (0) | 0/179 (0) | 0/179 (0) | 0/179 (0) | 0/179 (0) | 0/179 (0) | 0/179 (0) | 0/179 (0) | 0/179 (0) | 0/179 (0) |  |
| **Other** | 0/179 (0) | 0/179 (0) | 3/179 (1.63) | 0/179 (0) | 0/179 (0) | 0/179 (0) | 0/179 (0) | 0/179 (0) | 0/179 (0) | 0/179 (0) |  |

Table S30. Dermatological post-vaccination symptoms in children with comorbidities

|  | | **Local rash** | **Rash all over the body** | **Swelling of lymph nodes** | **Painful lymph nodes** | **Extensive redness of the skin** | **Other skin discolorations** | **Wheals/ hives** | **Blisters/ pustules** | **Petechiae** | **Hematoma** | **Dry skin** | **Eczema** | **Open skin areas/ wounds** | **Pruritus** | **Other complaints** |
| --- | --- | --- | --- | --- | --- | --- | --- | --- | --- | --- | --- | --- | --- | --- | --- | --- |
| **After First vaccination** | | 1/110 (0.91) | 0/110 (0) | 0/110 (0) | 1/110 (0.91) | 0/110 (0) | 0/110 (0) | 0/110 (0) | 0/110 (0) | 0/110 (0) | 0/110 (0) | 1/110 (0.91) | 2/110 (1.82) | 0/110 (0) | 0/110 (0) | 1/110 (0.91) |
| **After Second vaccination** | | 3/69 (4.35) | 0/69 (0) | 4/69 (5.80) | 4/69 (5.80) | 0/69 (0) | 0/69 (0) | 1/69 (1.45) | 0/69 (0) | 1/69 (1.45) | 0/69 (0) | 0/69 (0) | 0/69 (0) | 0/69 (0) | 0/69 (0) | 0/69 (0) |
| **Beginning & Duration** | |  |  |  |  |  |  |  |  |  |  |  |  |  |  |  |
| **Beginning (days after vaccination,mean±SD/N)** | 2±2/4 | 0±0/ 0 | 1.75± 0.96/ 4 | 1.4± 0.89/ 5 | 0±0/ 0 | 0±0/ 0 | 5±0 /1 | 0±0/ 0 | 10± 0/ 1 | 0±0/ 0 | 14±0/ 2 | 12.5±6.36/ 2 | 0±0/ 0 | 0±0/ 0 | 0.5± 0/ 1 |  |
| **Duration (days, mean±SD/N)** | 3.5± 3.32/ 4 | 0±0/ 0 | 5± 1.63/ 4 | 3.4± 1.52/ 5 | 0±0/ 0 | 0±0/ 0 | 1± 0/ 1 | 0±0/ 0 | 4± 0/ 1 | 0±0/ 0 | 7±0/ 2 | 4±4.24/ 2 | 0±0/ 0 | 0±0/ 0 | 3± 0/ 1 |  |
| **Aftermath** | |  |  |  |  |  |  |  |  |  |  |  |  |  |  |  |
| **Allergy medication/ pain medication** | 0/179 (0) | 0/179 (0) | 0/179 (0) | 0/179 (0) | 0/179 (0) | 0/179 (0) | 0/179 (0) | 0/179 (0) | 0/179 (0) | 0/179 (0) | 0/179 (0) | 0/179 (0) | 0/179 (0) | 0/179 (0) | 0/179 (0) |  |
| **Topical cremes** | 1/179 (0.56) | 0/179 (0) | 0/179 (0) | 0/179 (0) | 0/179 (0) | 0/179 (0) | 0/179 (0) | 0/179 (0) | 0/179 (0) | 0/179 (0) | 1/179 (0.56) | 2/179 (1.12) | 0/179 (0) | 0/179 (0) | 1/179 (0.56) |  |
| **Ambulatory** | 0/179 (0) | 0/179 (0) | 0/179 (0) | 0/179 (0) | 0/179 (0) | 0/179 (0) | 0/179 (0) | 0/179 (0) | 0/179 (0) | 0/179 (0) | 0/179 (0) | 0/179 (0) | 0/179 (0) | 0/179 (0) | 0/179 (0) |  |
| **Inpatient** | 0/179 (0) | 0/179 (0) | 0/179 (0) | 0/179 (0) | 0/179 (0) | 0/179 (0) | 0/179 (0) | 0/179 (0) | 0/179 (0) | 0/179 (0) | 0/179 (0) | 0/179 (0) | 0/179 (0) | 0/179 (0) | 0/179 (0) |  |
| **Mortality** | 0/179 (0) | 0/179 (0) | 0/179 (0) | 0/179 (0) | 0/179 (0) | 0/179 (0) | 0/179 (0) | 0/179 (0) | 0/179 (0) | 0/179 (0) | 0/179 (0) | 0/179 (0) | 0/179 (0) | 0/179 (0) | 0/179 (0) |  |
| **Other** | 0/179 (0) | 0/179 (0) | 0/179 (0) | 0/179 (0) | 0/179 (0) | 0/179 (0) | 0/179 (0) | 0/179 (0) | 0/179 (0) | 0/179 (0) | 0/179 (0) | 0/179 (0) | 0/179 (0) | 0/179 (0) | 0/179 (0) |  |

Table S31. Comparator analysis of post-vaccination symptoms in children with non-immunocompromising versus immunocompromising comorbidities

Immunocompromising comorbidities included rheumatological diseases, Primary Immunodeficiency and malignant diseases

|  | **Non-immuno-compromising comorbidity N=144 (%)** | **Immunocompromising comorbidity N=35 (%)** | **OR** | **95% CI** | **p** | **p_corrected_** |
| --- | --- | --- | --- | --- | --- | --- |
| **Local** | 97 (67.36) | 24 (68.57) | 1.057 | [0.4913 to 2.281] | >0.9999 | >0.9999 |
| **General** | 34 (23.61) | 8 (22.86) | 0.9586 | [0.4146 to 2.324] | >0.9999 | >0.9999 |
| **Musculoskeletal system** | 15 (10.42) | 2 (5.71) | 0.5212 | [0.1139 to 2.118] | 0.5316 | >0.9999 |
| **Gastrointestinal** | 12 (8.33) | 3 (8.57) | 1.031 | [0.2962 to 3.590] | >0.9999 | >0.9999 |
| **Otolaryngologic** | 8 (5.56) | 1 (2.86) | 0.5000 | [0.04384 to 3.520] | >0.9999 | >0.9999 |
| **Pulmonary** | 7 (4.86) | 1 (2.86) | 0.5756 | [0.04975 to 3.409] | >0.9999 | >0.9999 |
| **Cardiovascular** | 2 (1.39) | 0 (0.00) | NA^1^ | NA^1^ | >0.9999 | >0.9999 |
| **Neurological** | 21 (14.58) | 6 (17.14) | 1.212 | [0.4548 to 3.273] | 0.7923 | >0.9999 |
| **Psychological** | 8 (5.56) | 1 (2.86) | 0.5000 | [0.04384 to 3.520] | >0.9999 | >0.9999 |
| **Dermatological** | 12 (8.33) | 5 (14.29) | 1.833 | [0.6713 to 5.183] | 0.3326 | >0.9999 |

^1^ Not enough data for drawing conclusions
